# Effects of aspirin and omega-3 fatty acids on composite and subdomain scores from the NEI-VFQ-25 questionnaire: the ASCEND-Eye randomized controlled trial

**DOI:** 10.1186/s12886-024-03741-x

**Published:** 2024-11-05

**Authors:** Emily L. Sammons, Georgina Buck, Louise J. Bowman, William M. Stevens, Imen Hammami, Sarah Parish, Jane Armitage, on behalf of the ASCEND Study Collaborative Group

**Affiliations:** 1 Department of Population Health, Clinical Trial Service Unit and Epidemiological Studies Unit, University of Oxford, Nuffield, Richard Doll Building, Old Road Campus, Roosevelt Drive, Oxford, OX3 7LF UK; 2 Department of Population Health, Clinical Trial Service Unit and Epidemiological Studies Unit, University of Oxford, Nuffield Big Data Institute, Old Road Campus, Roosevelt Drive, Oxford, OX3 7LF UK

**Keywords:** Aspirin, Omega-3 fatty acids, NEI-VFQ-25, Randomized controlled trial, Diabetes

## Abstract

**Background:**

The double-blind, 2 × 2 factorial design, placebo-controlled ASCEND randomized trial compared the effects of 100 mg aspirin daily and, separately, 1 g omega-3 fatty acids (FAs) daily on the primary prevention of cardiovascular disease in 15,480 UK adults with diabetes. We report the effects of these randomized treatment allocations on scores derived from the National Eye Institute’s Visual Function Questionnaire-25 (NEI-VFQ-25) in a subset of participants involved in the ASCEND-Eye sub-study.

**Methods:**

Ordinal data from the NEI-VFQ-25 were analyzed using proportional odds regression methods. A common odds ratio with a 95% confidence interval was used to interpret the average effect size of randomization to each study treatment on composite and subdomain scores from the questionnaire.

**Results:**

Neither randomization to aspirin nor omega-3 FAs for 7.5 years significantly affected composite or subdomain scores from the NEI-VFQ-25.

**Conclusion:**

Applying the NEI-VFQ-25 in ASCEND-Eye represents one of the largest surveys of vision-targeted health-related quality of life in people with diabetes. Further observational analyses of these data are planned, to identify the clinical and demographic characteristics associated with lower composite and subdomain scores in a diabetic population.

**Trial registration:**

Eudract No. 2004–000991-15; Multicentre Research Ethics Committee Ref No. 03/8/087 (29th December 2003); ClinicalTrials.gov No. NCT00135226 (24th August 2005); ISRCTN No. ISRCTN60635500 (1st September 2005).

**Supplementary Information:**

The online version contains supplementary material available at 10.1186/s12886-024-03741-x.

## Introduction

ASCEND-Eye is an eye-health sub-study of the ASCEND (A Study of Cardiovascular Events iN Diabetes) double-blind, randomized, placebo-controlled trial of aspirin and omega-3 fatty acids (FAs) for the primary prevention of cardiovascular events in 15,480 UK adults, at least 40 years of age, with diabetes [1–4]. We have previously published the rationale, design and baseline characteristics [5] of this sub-study, as well as results showing a lack of any significant treatment effect on the primary outcome of referable diabetic retinopathy or maculopathy [6, 7]. We now present the results from pre-specified analyses of the sub-study’s secondary and tertiary efficacy outcomes, comparing the effects of aspirin versus placebo and, separately, omega-3 FAs versus placebo on composite and subdomain scores from the National Eye Institute’s Visual Function Questionnaire-25 (NEI-VFQ-25) [8].

## Research design and methods

### Trial oversight

Investigators from the Clinical Trial Service Unit at the University of Oxford designed and conducted ASCEND-Eye, overseen by the Trial Steering Committee. Members of the writing committee vouch for the sub-study’s faithfulness to the data analysis plan. The study adheres to CONSORT guidelines.

### Participants and procedures

The design of ASCEND has been described in detail elsewhere [1–4]. Briefly, the trial used mail-based methods to recruit and follow up 15,480 UK participants who were recruited between 2005 and 2011. They comprised men and women with type 1 or type 2 diabetes, who were at least 40 years of age, with no previous history of cardiovascular disease, no contraindication to the use of aspirin or omega-3 FAs, and no pre-existing life-limiting medical condition. Eligible participants were randomized in a 2 × 2 factorial double-blinded design, between 100 mg aspirin daily or matching placebo and, separately, 1 g omega-3 FA capsules (containing 460 mg eicosapentaenoic acid and 380 mg docosahexaenoic acid) daily or matching placebo. After randomization, participants were followed up using questionnaires sent every six months. All surviving participants who were on web- or mail-based follow-up at the end of the trial (31st July 2017) were sent the ASCEND-Eye Visual function questionnaire (VFQ) via the post (see supplementary materials) [8]. The VFQ consisted of two parts: a bespoke first page of questions which explicitly sought incident diagnoses of serious eye conditions, followed by the standard National Eye Institute Visual Function Questionnaire-25 (NEI-VFQ-25), which gathered information about the effects of visual impairment on activities of daily living and emotional well-being and has been reported to be a reliable and valid questionnaire across various chronic eye conditions [8]. Questionnaires were scanned and coded using commercial optical character recognition software supplemented by quality control software developed in-house for ASCEND. The participants’ signatures and dates of birth on every form were checked for consistency with those recorded on the ASCEND randomization form. Occasionally, resolving multiple or contradictory responses was also necessary by telephoning the participant.

### Outcomes

The analyses reported here were part of a more comprehensive programme of research conducted for the ASCEND-Eye sub-study, described in detail previously [5–7]. In this paper, we present the results from the pre-specified analyses of the secondary and tertiary efficacy outcomes, assessing the effects of randomization to aspirin compared with placebo and, separately, omega-3 FAs compared with placebo, on composite and subdomain scores from the NEI-VFQ-25, respectively.

### Statistical analysis

The data analysis plan was publicly available on the trial website (https://ascend.medsci.ox.ac.uk) before unblinding the ASCEND-Eye results. NEI-VFQ-25 composite and subdomain scores (general vision, ocular pain, near vision, distance vision, social function, mental health, role limitations, dependency, driving, colour vision and peripheral vision) were calculated using the scoring algorithm prescribed by the instrument developers (see Supplementary Materials), with a score of 0 representing the worst possible outcome and 100 representing perfect vision-specific quality of life [8]. As blinded preliminary analyses confirmed that the distribution of the composite score was negatively skewed, scores were grouped into a 5-point ordinal scale (≥ 90, 80–89, 70–79, 60–69 and < 60) and analyzed by proportional ordinal logistic regression [9]. This analysis method was chosen because there is simulated [10, 11] and empirical evidence [12, 13] that the method has greater power to detect differences in the treatment effect than if the data is dichotomized into a binary favourable versus unfavourable outcome. It assumes that treatment allocation has a similar effect on all pairs of grouped scores (i.e. the treatment effect between composite scores < 60 and ≥ 60 is the same as the treatment effect between composite scores < 70 and ≥ 70, etc.). Thus, a common odds ratio with 95% confidence interval (CI) was used to interpret the average effect size of randomization to aspirin versus placebo and, separately, omega-3 FAs versus placebo. The same approach was used to analyze the 11 subdomains, with the number and position of cut-points also based on a blinded preliminary assessment of the subdomain score distributions (Supplementary Table S1). To avoid multiple comparisons of the same data, we pre-specified that separate odds ratios between active and placebo groups at each response level would not be presented if the proportional odds assumption was violated. No formal adjustments for multiplicity were made for these secondary and tertiary analyses of ASCEND-Eye [5]. Therefore, two-tailed *P*-values of less than 0.05 and the 95% confidence interval should be interpreted with caution.

All analyses were carried out using SAS version 9.4 statistical software.

## Results

In total, 8,846 out of 11,301 participants eligible to receive the VFQ responded. Those who did not respond were slightly older and had a longer duration of diabetes (Supplementary Table S2). The baseline characteristics of VFQ responders were well-balanced between the randomized groups (Table 1). The mean (standard deviation) time from randomization to completing the VFQ was 8.6 (1.4) years; the mean (standard deviation) duration of trial treatment was 7.5 (1.4) years. Among those who returned a VFQ, the study average adherence to aspirin and omega-3 FAs was 79.3% and 88.4%, respectively, with similar proportions in the corresponding placebo arms (Table S3).

**Table 1:** Baseline characteristics by randomized treatment allocation in visual function questionnaire responders

Baseline characteristic	Aspirin randomization				Omega-3 FAs randomization				Overall (*n* = 8846)	
	**Active (*n* = 4447)**		**Placebo (*n* = 4399)**		**Active (*n* = 4417)**		**Placebo (*n* = 4429)**			
**Age at randomization (years)**										
Mean (SD)	62.4 ± 8.3		62.5 ± 8.3		62.4 ± 8.3		62.5 ± 8.3		62.5 ± 8.3	
**Sex**										
Male	2785	(62.6%)	2744	(62.4%)	2758	(62.4%)	2771	(62.6%)	5529	(62.5%)
Female	1662	(37.4%)	1655	(37.6%)	1659	(37.6%)	1658	(37.4%)	3317	(37.5%)
**Type of diabetes**^a^										
Type 1	270	(6.1%)	289	(6.6%)	288	(6.5%)	271	(6.1%)	559	(6.3%)
Type 2	4177	(93.9%)	4110	(93.4%)	4129	(93.5%)	4158	(93.9%)	8287	(93.7%)
**Duration of diabetes (years)**										
Median (IQR)	7 (3–12)		6 (3–12)		7 (3–12)		6 (3–12)		7(3–12)	
**Systolic blood pressure (mmHg)**^b^										
Mean (SD)	135.7 ± 15.1		135.9 ± 14.9		135.9 ± 15.1		135.7 ± 14.9		135.8 ± 14.9	
**Diastolic blood pressure (mmHg)**^b^										
Mean (SD)	77.3 ± 9.3		77.6 ± 9.1		77.5 ± 9.2		77.4 ± 9.1		77.4 ± 9.2	
**Body mass index (kg/m**^**2**^**)**^c^										
Mean (SD)	30.8 ± 6.3		30.4 ± 5.9		30.6 ± 6.1		30.6 ± 6.1		30.6 ± 6.1	
**Cigarette smoking**										
Current	264	(5.9%)	291	(6.6%)	278	(6.3%)	277	(6.3%)	555	(6.3%)
Former	1995	(44.9%)	1952	(44.4%)	1959	(44.4%)	1988	(44.9%)	3947	(44.6%)
Never	2139	(48.1%)	2104	(47.8%)	2131	(48.2%)	2112	(47.7%)	4243	(48.0%)
Unknown	49	(1.1%)	52	(1.2%)	49	(1.1%)	52	(1.2%)	101	(1.1%)
**Non-study medication**										
ACE-inhibitor or ARB	2559	(57.5%)	2556	(58.1%)	2597	(58.8%)	2518	(56.9%)	5115	(57.8%)
Aspirin use before screening	1598	(35.9%)	1559	(35.4%)	1582	(35.8%)	1575	(35.6%)	3157	(35.7%)
Thiazide or related diuretic	818	(18.4%)	821	(18.7%)	805	(18.2%)	834	(18.8%)	1639	(18.5%)
Calcium channel blocker	1074	(24.2%)	1014	(23.1%)	1040	(23.5%)	1048	(23.7%)	2088	(23.6%)
Statin	3424	(77.0%)	3345	(76.0%)	3356	(76.0%)	3413	(77.1%)	6769	(76.5%)
**Total cholesterol (mmol/L)**										
Mean (SD)	4.1 ± 0.9		4.2 ± 0.9		4.2 ± 0.9		4.1 ± 0.8		4.1 ± 0.9	
**HDL cholesterol (mmol/L)**										
Mean (SD)	1.3 ± 0.4		1.3 ± 0.4		1.3 ± 0.4		1.3 ± 0.4		1.3 ± 0.4	
**Non-HDL cholesterol (mmol/L)**										
Mean (SD)	2.9 ± 0.8		2.9 ± 0.8		2.9 ± 0.9		2.9 ± 0.8		2.9 ± 0.8	
**Glycosylated haemoglobin—HbA1c**										
IFCC (mmol/mol) mean (SD)	53.9 ± 12.1		54.2 ± 12.2		54.2 ± 12.1		53.9 ± 12.3		54.0 ± 12.2	
DCCT (%) mean (SD)	7.1 ± 1.1		7.1 ± 1.1		7.1 ± 1.1		7.1 ± 1.1		7.1 ± 1.1	
**CKD-EPI estimated GFR (ml/min/1.73m**^**2**^**)**^d^										
Mean (SD)	87.9 ± 19.6		87.7 ± 19.9		87.7 ± 20.2		87.8 ± 19.3		87.8 ± 19.8	
**Urinary albumin:creatinine ratio (mg/mmol)**										
Median (IQR)	0.50 (0.00–1.16)		0.49 (0.00–1.12)		0.50 (0.00–1.17)		0.50 (0.00–1.12)		0.50 (0.00–1.14)	
**Townsend Deprivation Index**^**e**^										
< -3	1537	(34.6%)	1564	(35.6%)	1574	(35.6%)	1527	(34.5%)	3101	(35.1%)
≥ -3 < 0	1830	(41.2%)	1758	(40.0%)	1762	(39.9%)	1826	(41.2%)	3588	(40.6%)
≥ 0 < 2	537	(12.1%)	534	(12.1%)	555	(12.6%)	516	(11.7%)	1071	(12.1%)
≥ 2 < 4	322	(7.2%)	303	(6.9%)	292	(6.6%)	333	(7.5%)	625	(7.1%)
≥ 4 < 6	161	(3.6%)	162	(3.7%)	162	(3.7%)	161	(3.6%)	323	(3.7%)
≥ 6	52	(1.2%)	66	(1.5%)	64	(1.4%)	54	(1.2%)	118	(1.3%)
Unknown	8	(0.2%)	12	(0.3%)	8	(0.2%)	12	(0.3%)	20	(0.2%)
**White Ethnicity**	4309	(96.9%)	4255	(96.7%)	4279	(96.9%)	4285	(96.7%)	8564	(96.8%)

Figures presented are counts with percentages unless otherwise stated. Percentages may not total 100 because of rounding

*ACE* Angiotensin converting enzyme, *ARB* Angiotensin receptor blocker, *DCCT* Diabetes Control and Complications Trial, *FAs* Fatty acids, *GFR* Glomerular Filtration Rate, *HDL* High-density lipoprotein, *IQR* Interquartile range, *IFCC* International Federation of Clinical Chemistry, *SD* Standard Deviation

^a^The presence of type 2 diabetes was based on a broad clinical definition involving the participant’s age when diagnosed with diabetes, the use of insulin within one year after diagnosis, and the body-mass index

^b^From blood and urine consent forms, generally before randomization

^c^The body-mass index (the weight in kilograms divided by the square of the height in metres) was based on values for height and weight the participants reported on their randomization questionnaires

^d^Calculated from blood cystatin c concentration using the CKD-EPI formula. (Inker LA, Schmid CH, Tighiouart H*, *et al*.* Estimating Glomerular Filtration Rate from Serum Creatinine and Cystatin C. *New England Journal of Medicine* 2012; 367(1): 20–9)

There was an analysis rule in ASCEND which stated that those with a below detectable threshold albumin component of their urinary albumin:creatinine ratio, would be recorded as zero. This applied to just over 25% of participants in the active arm of the aspirin randomization, and to just under 25% of participants in the placebo arm. Hence the interquartile range included zero

^e^Townsend scores of 0 represent an area with a UK-average level of deprivation, positive values indicate areas of above-average deprivation, and negative values indicate areas of below-average deprivation

**Fig. 1:**
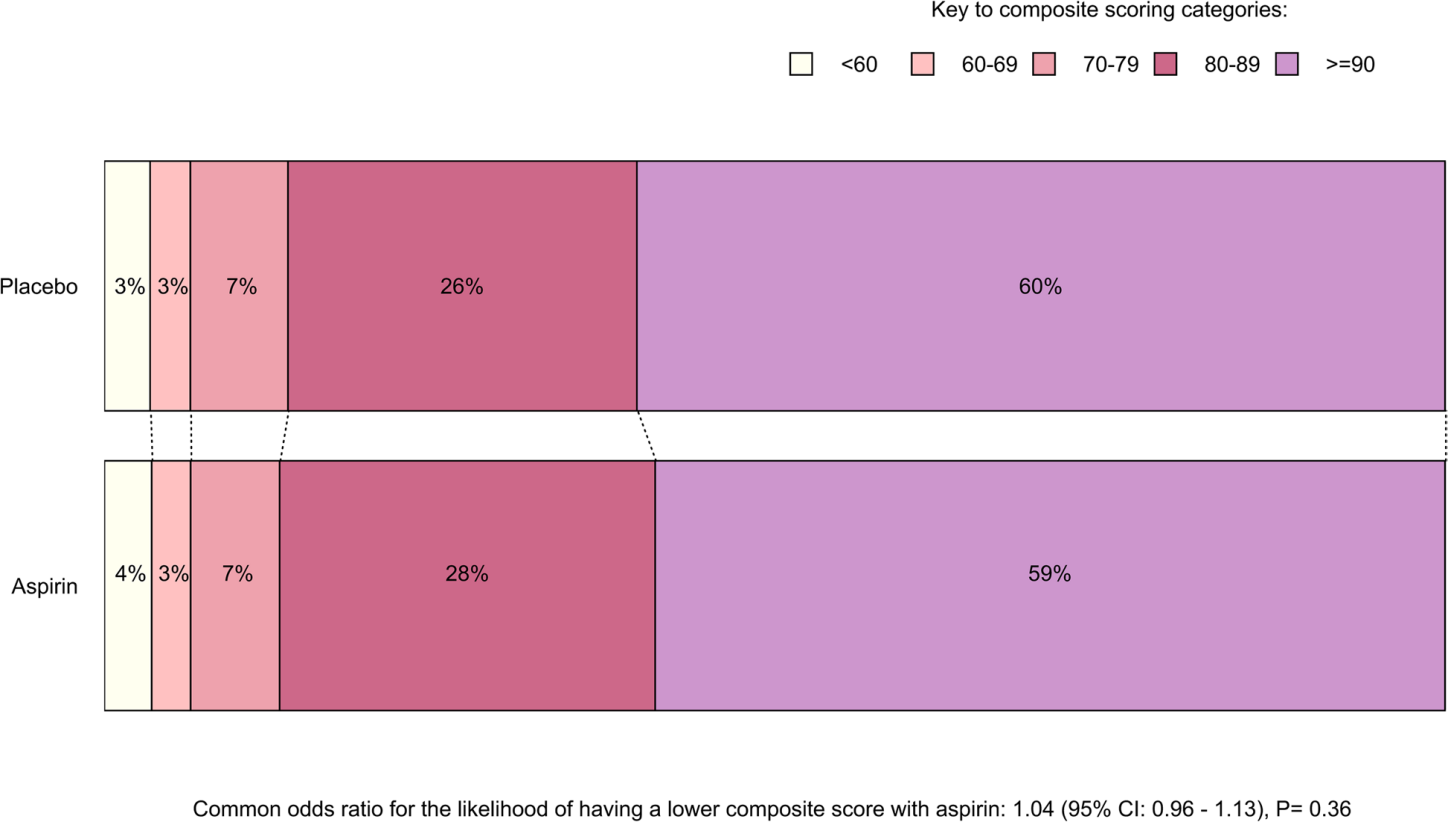
Composite scores from the NEI-VFQ-25 by Aspirin allocation

**Fig. 2:**
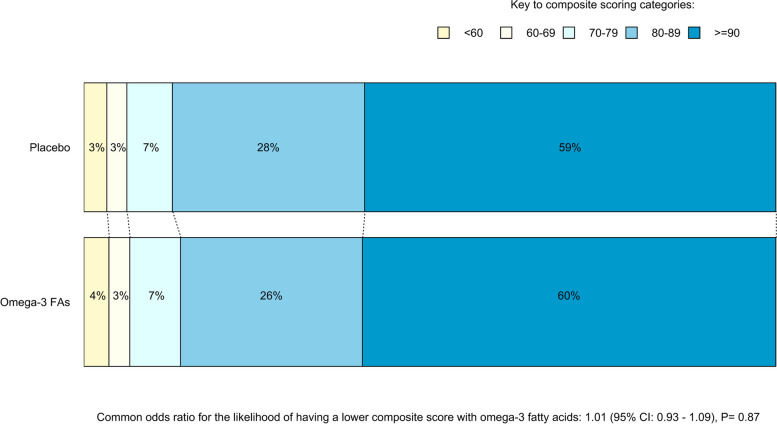
Composite scores from the NEI-VFQ-25 by Omega-3 fatty acids allocation

Of the 8,846 VFQ responders, 7 completed the page of questions on incident eye diagnoses but did not complete the NEI-VFQ-25, and 8,839 completed both parts. Mean composite and subdomain scores overall and by randomized treatment allocations are shown in Table 2. There was no statistically significant effect of either aspirin or omega-3 FAs on the composite scores. The common odds ratio for the likelihood of having a lower composite score with randomization to aspirin versus placebo was 1.04 (95% CI 0.96–1.13; *P* = 0.36; Fig. 1), and for omega-3 FAs versus placebo, was 1.01 (95% CI 0.93–1.09; *P* = 0.87; Fig. 2). In exploratory analyses, the proportional effects of aspirin and, separately, omega-3 FAs on NEI-VFQ-25 score did not vary significantly by other treatment assignment (*P* = 0.75 for interaction in the aspirin model and *P* = 0.84 in the omega-3 FAs model). The effect of randomized treatment allocations on the odds of lower subdomain scores were also non-significant for most of the 11 subdomains (Figures S1-S22 in the supplementary materials). Although the *p*-value for a greater likelihood of having a less-than-perfect score for the vision-specific dependency subdomain by omega-3 FAs allocation was 0.04 (odds ratio 1.16; 95% CI 1.01–1.33; Figure S16), this could be a chance finding given the number of comparisons analyzed. The assumption of proportional odds was upheld in every model except for the driving subdomain scores by omega-3 FAs allocation (*P* = 0.01, Figure S18). Separate analyses to calculate an odds ratio at each data cut-point of this subdomain have not been performed, as per the pre-specified data analysis plan.

**Table 2:** Average composite NEI-VFQ-25 scores—overall, by subdomain and by randomized treatment allocation

	**Aspirin randomization**		**Omega-3 FAs randomization**		**Overall**
	**Active**	**Placebo**	**Active**	**Placebo**	
	***n* = 4444**	***n* = 4395**	***n* = 4415**	***n* = 4424**	***n* = 8839**
**Composite score**	87.9 (11.1)	88.2 (11.0)	87.9 (11.3)	88.2 (10.8)	88.1 (11.1)
**Subdomain score**					
	***n* = 4305**	***n* = 4242**	***n* = 4288**	***n* = 4259**	***n* = 8547**
General health^a^	53.9 (21.2)	54.2 (21.7)	54.1 (21.1)	54.1 (21.8)	54.1 (21.4)
	***n* = 4431**	***n* = 4381**	***n* = 4403**	***n* = 4409**	***n* = 8812**
General vision	78.4 (13.7)	78.4 (13.4)	78.4 (13.7)	78.3 (13.7)	78.4 (13.5)
	***n* = 4444**	***n* = 4394**	***n* = 4414**	***n* = 4424**	***n* = 8838**
Ocular pain	91.3 (14.1)	91.7 (13.8)	91.4 (14.1)	91.6 (13.8)	91.5 (13.9)
	***n* = 4433**	***n* = 4382**	***n* = 4402**	***n* = 4413**	***n* = 8815**
Near activities	88.0 (15.9)	88.1 (15.9)	87.8 (16.3)	88.3 (15.5)	88.1 (15.9)
	***n* = 4433**	***n* = 4381**	***n* = 4404**	***n* = 4410**	***n* = 8814**
Distance activities	91.9 (13.9)	92.1 (13.6)	91.9 (14.0)	92.2 (13.5)	92.1 (13.8)
	***n* = 4429**	***n* = 4381**	***n* = 4401**	***n* = 4409**	***n* = 8810**
Social functioning	96.5 (11.0)	96.5 (10.9)	96.3 (11.4)	96.7 (10.5)	96.5 (10.9)
	***n* = 4441**	***n* = 4392**	***n* = 4413**	***n* = 4420**	***n* = 8833**
Mental health	90.4 (14.8)	90.7 (14.5)	90.2 (15.0)	90.9 (14.3)	90.5 (14.7)
	***n* = 4389**	***n* = 4345**	***n* = 4361**	***n* = 4373**	***n* = 8734**
Role dependency	91.2 (17.6)	91.2 (17.7)	91.0 (17.9)	91.5 (17.3)	91.2 (17.6)
	***n* = 4421**	***n* = 4372**	***n* = 4390**	***n* = 4403**	***n* = 8793**
Vision-specific dependency	96.8 (12.1)	97.0 (11.5)	96.8 (12.1)	97.1 (11.6)	96.9 (11.8)
	***n* = 3215**	***n* = 3201**	***n* = 3221**	***n* = 3195**	***n* = 6416**
Driving	87.9 (20.7)	88.9 (19.1)	87.9 (20.8)	88.8 (18.9)	88.4 (19.9)
	***n* = 4392**	***n* = 4350**	***n* = 4365**	***n* = 4377**	***n* = 8742**
Colour vision	97.2 (10.7)	97.3 (10.3)	97.3 (10.5)	97.2 (10.6)	97.3 (10.5)
	***n* = 4419**	***n* = 4361**	***n* = 4394**	***n* = 4386**	***n* = 8780**
Peripheral vision	93.3 (15.2)	93.3 (15.2)	93.3 (15.2)	93.3 (15.2)	93.3 (15.2)

Figures are presented as mean (standard deviation). The number of participants who gave non-missing answers and contributed to the composite and subdomain score analyses is shown in the row above the mean and standard deviation results

*FAs* Fatty acids

^a^The general health subdomain is not included in the calculation of composite scores from the NEI-VFQ-25. The instrument developers intend the composite and subdomain scores to be interpreted alongside the general health score and hence, they are displayed in the table. Comparisons of the responses to this question by randomized treatment allocation were conducted on a post-hoc basis and are shown in the supplementary materials

## Discussion

In this ASCEND-Eye sub-study of a large randomized placebo-controlled trial, neither randomization to aspirin nor omega-3 FAs for a mean of 7.5 years had any significant effect on composite scores derived from the NEI-VFQ-25, nor on any of its subdomains at a mean of 8.6 years from randomization. Scores obtained from the NEI-VFQ-25 are strongly and independently correlated to visual acuity [8]. The latter serves as an objective indicator of macular function, but it may not reflect how retinal diseases affect other dimensions of vision, including contrast sensitivity, colour perception and stereoscopic vision [14]. Therefore, the NEI-VFQ-25 has been recommended by regulators as a more holistic endpoint and for vision-targeted cost-utility analyses in ophthalmology trials, such as ASCEND-Eye [15]. Few previous studies of aspirin or omega-3 FAs in diabetes have investigated their effects on eye disease and none have looked at their effects on self-reported vision-targeted quality of life scores derived from the NEI-VFQ-25 or similar questionnaires. However, the average composite and subdomain scores in ASCEND-Eye were consistent with those reported in observational studies of individuals with diabetes [14, 16–18].

Our study has some limitations. The distribution of scores from the NEI-VFQ-25 were very negatively skewed towards the highest possible values, both overall and in the subdomains. Therefore, showing between-group differences with this instrument would have required both a large treatment effect and a potential for rapid deterioration of retinal disease during the follow-up period. In ASCEND-Eye, very few participants had severe retinal disease, and both conditions that were of interest, diabetic retinopathy and age-related macular degeneration, are known to progress slowly compared to the duration of follow-up. Since it was not possible to send the NEI-VFQ-25 to those on third-party follow-up at the end of ASCEND, those who completed the questionnaire were slightly younger (Table S1), probably less frail and probably with better eyesight than those who were not sent it, making it more difficult to detect any effects. The absence of any treatment effect on the primary and secondary outcome comparisons [6, 7], may explain and is consistent with the fact that there were no significant treatment effects on the NEI-VFQ-25 composite or subdomain scores over the follow-up period. Finally, the generalizability of the results in a real-world setting may be limited by a lack of ethnic diversity and the relative under-representation of women in ASCEND.

## Conclusion

Overall, the application of the NEI-VFQ-25 in ASCEND-Eye represents one of the largest surveys of vision-targeted health-related quality of life in people with diabetes to date. No effect of aspirin or omega-3 FAs on NEI-VFQ-25 scores was seen. Some observational analyses are planned to further investigate these data, which may identify the clinical and demographic characteristics associated with lower composite and subdomain scores in a diabetic population. This will include an assessment of the associations between diabetic retinopathy severity and NEI-VFQ-25 scores.

## Supplementary Information

Supplementary Material 1.

## Data Availability

All requests for data sharing should be addressed to Professors Jane Armitage and Louise Bowman, Co-Principal Investigators of ASCEND, and will be handled in line with the data access and sharing policy of the Nuffield Department of Population Health, University of Oxford (www.ndph.ox.ac.uk/about/data-access-policy).

## Abbreviations

ASCEND: A Study of Cardiovascular Events iN Diabetes
NEI-VFQ-25: National Eye Institute Visual Function Questionnaire-25

## References

[1] Aung T, Haynes R, Barton J, et al. Cost-effective recruitment methods for a large randomised trial in people with diabetes: a Study of Cardiovascular Events iN Diabetes (ASCEND). Trials. 2016;17(1):286. 27296091 10.1186/s13063-016-1354-9 PMC4907276

[2] ASCEND Collaborative Group. ASCEND: A Study of Cardiovascular Events iN Diabetes: characteristics of a randomized trial of aspirin and of omega-3 fatty acid supplementation in 15,480 people with diabetes. Am Heart J. 2018;198:135–44. 29653635 10.1016/j.ahj.2017.12.006 PMC5971211

[3] ASCEND Collaborative Group. Effects of aspirin for primary prevention in persons with diabetes mellitus. N Engl J Med. 2018;379(16):1529–39. 30146931 10.1056/NEJMoa1804988

[4] Bowman L, Mafham M, Wallendszus K, et al. Effects of n-3 fatty acid supplements in diabetes mellitus. N Engl J Med. 2018;379(16):1540–50. 30146932 10.1056/NEJMoa1804989

[5] Sammons E, Bowman L, Stevens W, et al. ASCEND-Eye: rationale, design and baseline characteristics for a sub-study of the ASCEND randomised trial, exploring the effects of aspirin and omega-3 fatty acids on diabetic retinopathy and age-related macular degeneration. Contemp Clin Trials Commun. 2023;35:101184. 37745288 10.1016/j.conctc.2023.101184 PMC10517364

[6] Sammons EL, Buck G, Bowman LJ, et al. ASCEND-Eye: effects of omega-3 fatty acids on diabetic retinopathy. Ophthalmology. 2024;131(5):526–33. 38052385 10.1016/j.ophtha.2023.11.030

[7] Sammons EL, Buck G, Bowman LJ, et al. ASCEND-Eye: effects of aspirin on diabetic retinopathy. Ophthalmology. 2024;131(7):771–9. 38237868 10.1016/j.ophtha.2024.01.018

[8] Mangione CM, Lee PP, Gutierrez PR, et al. Development of the 25-list-item National Eye Institute Visual Function Questionnaire. Arch Ophthalmol. 2001;119(7):1050–8. 11448327 10.1001/archopht.119.7.1050

[9] Agresti A. Logistic regression models using cumulative logits. Analysis of ordinal categorical data. 2nd ed. Wiley Series in Probability and Statistics; 2012. p. 44–87. Available from: https://download.e-bookshelf.de/download/0000/6452/64/L-G-0000645264-0003879127.pdf. Accessed 24 Oct 2024.

[10] McHugh GS, Butcher I, Steyerberg EW, et al. A simulation study evaluating approaches to the analysis of ordinal outcome data in randomized controlled trials in traumatic brain injury: results from the IMPACT Project. Clin Trials. 2010;7(1):44–57. 20156956 10.1177/1740774509356580

[11] Valenta Z, Pitha J, Poledne R. Proportional odds logistic regression–effective means of dealing with limited uncertainty in dichotomizing clinical outcomes. Stat Med. 2006;25(24):4227–34. 16929469 10.1002/sim.2678

[12] Bath PM, Gray LJ, Collier T, Pocock S, Carpenter J. Can we improve the statistical analysis of stroke trials? Statistical reanalysis of functional outcomes in stroke trials. Stroke. 2007;38(6):1911–5. 17463316 10.1161/STROKEAHA.106.474080

[13] Roozenbeek B, Lingsma HF, Perel P, et al. The added value of ordinal analysis in clinical trials: an example in traumatic brain injury. Crit Care. 2011;15(3):R127. 21586148 10.1186/cc10240 PMC3218993

[14] Klein R, Moss SE, Klein BE, Gutierrez P, Mangione CM. The NEI-VFQ-25 in people with long-term type 1 diabetes mellitus: the Wisconsin Epidemiologic Study of Diabetic Retinopathy. Arch Ophthalmol. 2001;119(5):733–40. 11346401 10.1001/archopht.119.5.733

[15] European Medicines Agency. EU regulatory workshop – ophthalmology: visual function endpoints in clinical trials. (2012). http://www.ema.europa.eu/docs/en_GB/document_library/Report/2012/09/WC500131815.pdf. Accessed 21 Mar 2018.

[16] Mazhar K, Varma R, Choudhury F, McKean-Cowdin R, Shtir CJ, Azen SP. Severity of diabetic retinopathy and health-related quality of life: the Los Angeles Latino Eye Study. Ophthalmology. 2011;118(4):649–55. 21035872 10.1016/j.ophtha.2010.08.003 PMC3070833

[17] Trento M, Passera P, Trevisan M, et al. Quality of life, impaired vision and social role in people with diabetes: a multicenter observational study. Acta Diabetol. 2013;50(6):873–7. 23526056 10.1007/s00592-013-0470-1

[18] Trento M, Durando O, Lavecchia S, et al. Vision related quality of life in patients with type 2 diabetes in the EUROCONDOR trial. Endocrine. 2017;57(1):83–8. 27628581 10.1007/s12020-016-1097-0

