# Supplementary material for: Effects of aspirin and omega-3 fatty acids on composite and subdomain scores from the NEI-VFQ-25 questionnaire: the ASCEND-Eye randomized controlled trial
Source: BMC Ophthalmol. 2024 Nov 5;24:481. doi: 10.1186/s12886-024-03741-x (PMC11536542; doi:10.1186/s12886-024-03741-x)
Supplement: Supplementary file 1 — Supplementary Material 1. [file 12886_2024_3741_MOESM1_ESM.docx]

**Supplementary Appendix 1**

This supplement contains the following items:

[ASCEND Study Collaborative Group 3](#_Toc162296075)

[ASCEND-Eye Visual Function Questionnaire 6](#_Toc162296076)

[Calculation of NEI-VFQ-25 composite and subdomain scores 7](#_Toc162296077)

[Table S1: Data Cut-Points for NEI-VFQ-25 Composite and Vision-Targeted Subdomain Scoring Categories 9](#_Toc162296078)

[Table S2 Baseline Characteristics of Visual Functioning Questionnaire Responders and Non-Responders by Eligibility to be sent the Questionnaire 10](#_Toc162296079)

[Table S3 Reported Definite or Probable Adherence with Study Treatment Stratified by Years Post-Randomisation 13](#_Toc162296080)

[Figure S1 General Vision Subdomain Score from the NEI-VFQ-25 by Aspirin Allocation 14](#_Toc162296081)

[Figure S2 General Vision Subdomain Score from the NEI-VFQ-25 by Omega-3 Fatty Acids Allocation 14](#_Toc162296082)

[Figure S3 Ocular Pain Subdomain Score from the NEI-VFQ-25 by Aspirin Allocation 15](#_Toc162296083)

[Figure S4 Ocular Pain Subdomain Score from the NEI-VFQ-25 by Omega-3 Fatty Acids Allocation 15](#_Toc162296084)

[Figure S5 Near Activities Subdomain Score from the NEI-VFQ-25 by Aspirin Allocation 16](#_Toc162296085)

[Figure S6 Near Activities Subdomain Score from the NEI-VFQ-25 by Omega-3 Fatty Acids Allocation 16](#_Toc162296086)

[Figure S7 Distance Activities Subdomain Score from the NEI-VFQ-25 by Aspirin Allocation 17](#_Toc162296087)

[Figure S8 Distance Activities Subdomain Score from the NEI-VFQ-25 by Omega-3 Fatty Acids Allocation 17](#_Toc162296088)

[Figure S9 Social Functioning Subdomain Score from the NEI-VFQ-25 by Aspirin Allocation 18](#_Toc162296089)

[Figure S10 Social Functioning Subdomain Score from the NEI-VFQ-25 by Omega-3 Fatty Acids Allocation 18](#_Toc162296090)

[Figure S11 Mental Health Subdomain Score from the NEI-VFQ-25 by Aspirin Allocation 19](#_Toc162296091)

[Figure S12 Mental Health Subdomain Score from the NEI-VFQ-25 by Omega-3 Fatty Acids Allocation 19](#_Toc162296092)

[Figure S13 Role Dependency Subdomain Score from the NEI-VFQ-25 by Aspirin Allocation 20](#_Toc162296093)

[Figure S14 Role Dependency Subdomain Score from the NEI-VFQ-25 by Omega-3 Fatty Acids Allocation 20](#_Toc162296094)

[Figure S15 Vision-Specific Dependency Subdomain Score from the NEI-VFQ-25 by Aspirin Allocation 21](#_Toc162296095)

[Figure S16 Vision-Specific Dependency Subdomain Score from the NEI-VFQ-25 by Omega-3 Fatty Acids Allocation 21](#_Toc162296096)

[Figure S17 Driving Subdomain Score from the NEI-VFQ-25 by Aspirin Allocation 22](#_Toc162296097)

[Figure S18 Driving Subdomain Score from the NEI-VFQ-25 by Omega-3 Fatty Acids Allocation 22](#_Toc162296098)

[Figure S19 Colour Vision Subdomain Score from the NEI-VFQ-25 by Aspirin Allocation 23](#_Toc162296099)

[Figure S20 Colour Vision Subdomain Score from the NEI-VFQ-25 by Omega-3 Fatty Acids Allocation 23](#_Toc162296100)

[Figure S21 Peripheral Vision Subdomain Score from the NEI-VFQ-25 by Aspirin Allocation 24](#_Toc162296101)

[Figure S22 Peripheral Vision Subdomain Score from the NEI-VFQ-25 by Omega-3 Fatty Acids Allocation 24](#_Toc162296102)

[Figure S23 General Health Subdomain Score from the NEI-VFQ-25 by Aspirin Allocation 25](#_Toc162296103)

[Figure S24 General Health Subdomain Score from the NEI-VFQ-25 by Omega-3 Fatty Acids Allocation 25](#_Toc162296104)

[References 26](#_Toc162296105)

## ASCEND Study Collaborative Group

**Writing Committee**

Emily Sammons (*Corresponding author)*, Louise Bowman, William Stevens, Georgina Buck, Imen Hammami, Sarah Parish, Jane Armitage

**Steering Committee**

*Chairman:* R Collins, *Study coordinator:* J Armitage; *Clinical coordinator:* L Bowman; *Statisticians:* S Parish, R Peto; *Administrative coordinator:* J Barton; *Lay member:* D Simpson; *Other members:* A Adler, T Aung, C Baigent, HJ Bodansky, A Farmer, R Haynes, R McPherson, M Mafham, HAW Neil, N Samani, P Sleight, P Weissberg.

**Data Monitoring Committee**

*Chair:* P Sandercock, *Members:* H Gerstein, R Gray, C Hennekens.

**Coordinating Office (Clinical Trial Service Unit, Nuffield Department of Population Health, University of Oxford):**

*Administration and support:* J Barton, L Fletcher, K Murphy (coordinators); S Hurley, R Lee, S Pickworth, M Willett, M Wincott.

*Clinical support and adjudication:* J Armitage, L Bowman, M Mafham, E Sammons

*Statistics and computing:* M Lay, S Parish; G Buck, A Murawska, W Stevens, K Wallendszus, A Young, I Hammami

*Research support:* K Melham, G Brown, J Latham-Mollart, A Brewer

**Collaborators**

*Ophthalmologists:* P Scanlon^1^, P Patel^2^

*Public Health England* (now the UK Health Security Agency): M Olson^3^

*Public Health Wales^4^:* J Kay, S Banerjee, L Evans, A Davies, M Griffiths, H Clayton

*Health Intelligence Ltd^5^:* P Kirby. M Pennington^8^, D Clarke

*Northgate Ltd^6^:* J Anslow, A Hallam, J Witts, S Egan, A Wharton

*Cheshire DESP^7^:* A Sachdev, A Derbyshire, E Williamson, K Hepplestone

*East and North Hertfordshire DESP^8^:* S Mithra, S Oliver, P Wiatrak-Olszewska

*Greater Nottingham DESP^9^:* T Gazis, K Alvey, E Wu

*Humber DESP^10^:* H Cook, N Gregory, P Parkinson

*North East London DESP^11^:* J Anderson, L Bolter

*North Nottinghamshire DESP^12^:* P Maharajan, R McFee, L Allsop, D Sowter, D Hodgson

*North Yorkshire DESP^13^:* J Thow, J Featonby, R Furnival

*Oxfordshire DESP^14^:* P Scanlon, H Lipinski, H Benjamin, T McAfee

*South East Sussex DESP^15^:* E Payne, L Still

**Funding**

The Macular Society^16^

British Heart Foundation^17^

1. Department of Ophthalmology, Gloucestershire Hospitals NHS Foundation Trust, Cheltenham General Hospital, Sandford Road, Cheltenham, GL53 7AN
2. Moorfields Eye Hospital, 162 City Road, London, EC1V 2PD
3. Health Information Consulting Ltd, Low Barn, Homestall Lane, Faversham, Kent, ME13 8UT
4. Public Health Wales Research and Evaluation Division, Knowledge Directorate, Floor 5, 2 Capital Quarter, Tyndall Street, Cardiff, CF10 4BZ
5. InHealth Intelligence, Unity House, Road 5, Winsford Industrial Estate, Winsford, Cheshire, CW7 3RB
6. Northgate Public Services (UK) Ltd, Crome Lea Business park, Madingley road, Coton, CB23 7PH
7. Cheshire Diabetic Eye Screening Programme, Eagle Bridge Health and Wellbeing Centre, Dunwoody Way, Crewe, CW1 3AW
8. East and North Hertfordshire Diabetic Eye Screening Programme, Administration Centre H4, Hertford County Hospital, North Road, Hertford, SG14 1LP
9. Greater Nottinghamshire Diabetic Eye Screening Programme, 2nd Floor, Ropewalk House, 113 The Ropewalk, Nottingham, NG1 5DU
10. Humber Diabetic Eye Screening Programme, Alderson House, Hull Royal Infirmary, Hull, HU3 2JZ
11. North East London Diabetic Eye Screening Programme, The Eye Screening Centre, Homerton University Hospital, Homerton Row, Hackney, London, E9 6SR
12. North Nottinghamshire Diabetic Eye Screening Programme, Kings Mill Hospital, Trust Admin Building - Level 2, Mansfield Road, Sutton In Ashfield, Notts, NG17 4JL
13. North Yorkshire Diabetic Eye Screening Programme, 2 Cayley Court, George Cayley Drive, Clifton Moor, York, YO30 4WH
14. Oxfordshire Diabetic Eye Screening Programme, Level 0, West Wing, John Radcliffe Hospital, Headley Way, Headington, Oxford, OX3 9DU
15. South East Sussex Diabetic Eye Screening Programme, Bexhill Hospital, East Sussex Healthcare NHS Trust, Hollier's Hill, Bexhill-on-Sea, TN40 2DZ
16. Macular Society, Crown Chambers, South Street, Andover, SP10 2BN
17. British Heart Foundation, Greater London House, 5th Floor, 180 Hampstead Road, London, UK, NW1 7AW

##

## ASCEND-Eye Visual Function Questionnaire


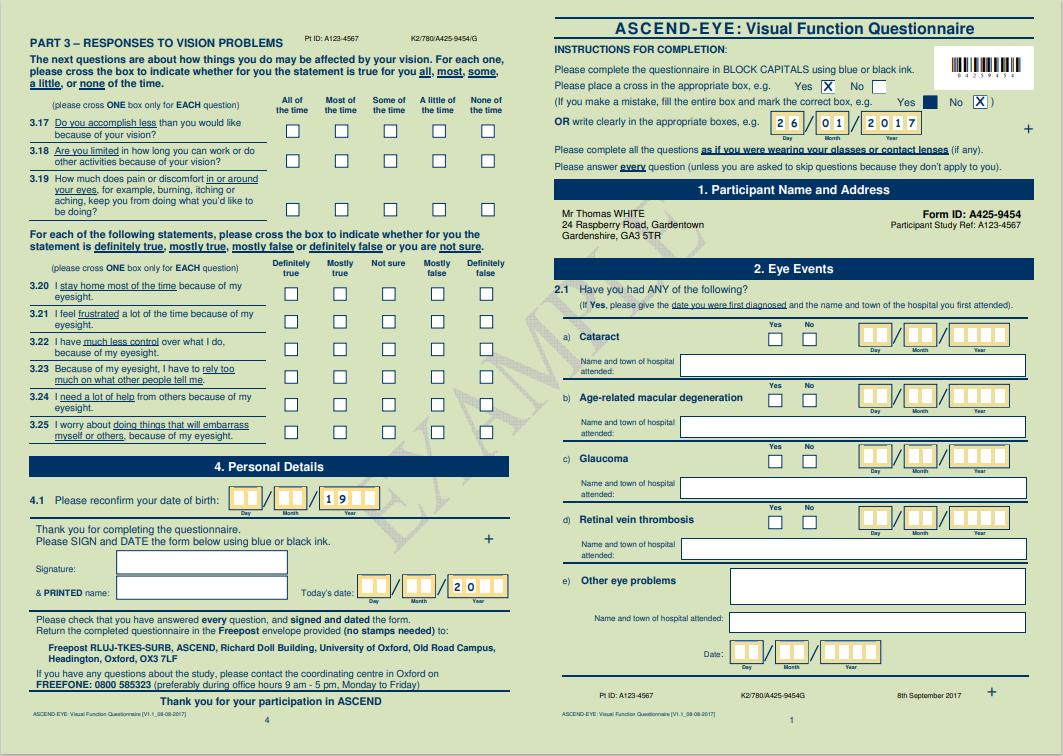


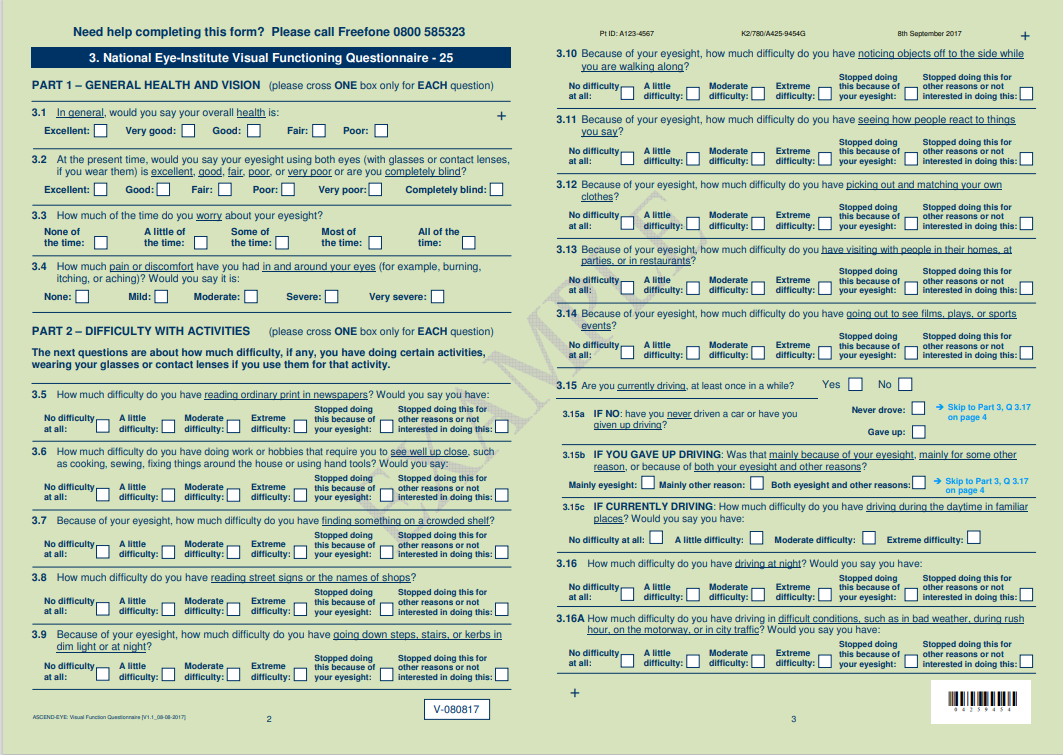


Calculation of NEI-VFQ-25 composite and subdomain scores

The NEI-VFQ-25 subdomain and composite scores were calculated using the “NEI-VFQ-25 Scoring Algorithm – August 2000” ^1^ as follows: Step 1: Numeric values from the survey were recoded following the scoring rules shown in table A. All items were converted to a 0 to 100 scale where higher scores represented better functioning. In this format, scores represent the achieved percentage of the possible total, e.g. a score of 25 represents 25% of the highest possible score.

Step 2: Items within each subdomain were averaged together. Table B indicates which items contributed to each subdomain. Items left blank (missing data) were not considered when calculating the subdomain scores. Subdomains with at least one item answered were used to generate a subdomain score. Hence, scores represented the average for all items in the subdomain that the respondent answered.

The subdomain scores were then averaged to calculate an overall composite score, excluding the general health rating question (Q1). By averaging subdomain scores rather than individual items, equal weight is given to each visual domain, whereas averaging the items would give more weight to domains with more items.

| **Table A: Recoding of responses (analysis step 1)** | |  |
| --- | --- | --- |
| **Question Number** | **Change response category** | **Recoded value** |
| 1,3,4,15c | 1 | 100 |
|  | 2 | 75 |
|  | 3 | 50 |
|  | 4 | 25 |
|  | 5 | 0 |
| 2 | 1 | 100 |
|  | 2 | 80 |
|  | 3 | 60 |
|  | 4 | 40 |
|  | 5 | 20 |
|  | 6 | 0 |
| 5,6,7,8,9,10,11, 12, 13, 14, 16, 16a, | 1 | 100 |
|  | 2 | 75 |
|  | 3 | 50 |
|  | 4 | 25 |
|  | 5 | 0 |
|  | 6 | * |
| 17, 18, 19, 20, 21, 22, 23, 24, 25 | 1 | 0 |
|  | 2 | 25 |
|  | 3 | 50 |
|  | 4 | 75 |
|  | 5 | 100 |

*Response choice “6” indicates that the person does not perform the activity because of non-vision-related problems. If this choice is selected, the item is coded as missing.

**Table B: Averaging of questions to generate NEI-VFQ-25 subdomains (analysis step 2)**

| **Scale** | **Number of questions** | **Questions to be averaged after recoding** |
| --- | --- | --- |
| General health | 1 | 1 |
| General vision | 1 | 2 |
| Ocular pain | 2 | 4, 19 |
| Near activities | 3 | 5, 6, 7 |
| Distance activities | 3 | 8, 9. 14 |
| Vision-specific: social functioning | 2 | 11,13 |
| Vision-specific: mental health | 4 | 3, 21, 22, 25 |
| Vision-specific: role difficulties | 2 | 17, 18 |
| Vision-specific: dependency | 3 | 20, 23, 24 |
| Driving * | 3 | 15c, 16, 16a |
| Colour vision | 1 | 12 |
| Peripheral vision | 1 | 10 |

* Q15c has four response levels, which are expanded to a 5-level response using Q15b:

- If 15b = 1, then 15c should be recoded to 0
- If 15b = 2, then 15c should be recoded as missing
- If 15b = 3, then 15c should be recoded as missing.

Questions 15c, 16 and 16a represent the only questions included in the driving subdomain score. However, participants navigate to Q15c via three screening questions to clarify whether they are currently driving. Where there was missing data or where a nonsensical combination of answers was given for questions 15 to 16a, ASCEND-Eye applied the following rules:

Analysis of Q15c was restricted to those who either indicated in Q15 that they were current drivers or indicated in Q15a or Q15b that they have given up driving. Questions 16 and 16a may be answered by anyone who has ever driven, including those who are currently driving (as in question 15) and those who have previously driven but gave up (question 15a). Questions 16 and 16a offer a second opportunity to indicate that an individual has stopped driving at night or in adverse weather conditions, respectively, due to visual impairment or other reasons. Therefore, individuals were included in analyses of questions 16 and 16a if an answer had been given, irrespective of their responses to questions 15, 15b or 15c, but those who responded to question 15a that they have never driven were excluded.

## Table S1: Data Cut-Points for NEI-VFQ-25 Composite and Vision-Targeted Subdomain Scoring Categories

| **NEI-VFQ-25 Component** | **Ordinal Category*** |
| --- | --- |
|  |  |
| **Composite score** | ≥90, 80-89, 70-79, 60-69 and <60 |
|  |  |
| **Subdomain score** |  |
| General Vision | 100,80-99,60-79 and <60 |
| Ocular Pain | ≥90,80-89, 70-79,60-69 and <60 |
| Near Activities | 100, 90-99, 80-89, 70-79 and <70 |
| Distance Activities | 100, 90-99, 80-89, 70-79 and <70 |
| Social Functioning | ≥90, 80-89, 70-79 and <70 |
| Mental Health | ≥90, 80-89, 70-79, 60-69 and <60 |
| Role Dependency | ≥90, 80-89, 70-79 and <70 |
| Vision-Specific Dependency | 100, <100 |
| Driving | 100, 80-99, 60-79 and <60 |
| Colour Vision | 100,75-99 and <75 |
| Peripheral Vision | 100,75-99 and <75 |
| General Health† | 100, 75-99, 50-74, 25-49 and <25 |

*Data cut points were chosen on the basis of preliminary blinded assessment of the distribution of scores

†Analyses of the General Health subdomain were conducted post-hoc.

## Table S2 Baseline Characteristics of Visual Functioning Questionnaire Responders and Non-Responders by Eligibility to be sent the Questionnaire

| **Baseline Characteristic** | **Eligible to be sent the VFQ (n=11,301)** | | | | **Ineligible to be sent the VFQ**  **(n=4179)** | |
| --- | --- | --- | --- | --- | --- | --- |
|  | **Responders**  **(n=8846)*** | | **Non-responders (n=2455)** | |  |  |
|  |  |  |  |  |  |  |
| **Age at randomisation (years)** |  |  |  |  |  |  |
| Mean (SD) | 62.5±8.3 | | 62.8±9.0 | | 65.2±10.7 | |
| <60 | 3339 | (37.7%) | 901 | (36.7%) | 1350 | (32.3%) |
| ≥60<70 | 3860 | (43.6%) | 1024 | (41.7%) | 1363 | (32.6%) |
| ≥70 | 1647 | (18.6%) | 530 | (21.6%) | 1466 | (35.1%) |
|  |  |  |  |  |  |  |
| **Sex** |  |  |  |  |  |  |
| Male | 5529 | (62.5%) | 1550 | (63.1%) | 2605 | (62.3%) |
| Female | 3317 | (37.5%) | 905 | (36.9%) | 1574 | (37.7%) |
|  |  |  |  |  |  |  |
| **Type of diabetes** |  |  |  |  |  |  |
| Type 1 | 559 | (6.3%) | 138 | (5.6%) | 214 | (5.1%) |
| Type 2 | 8287 | (93.7%) | 2317 | (94.4%) | 3965 | (94.9%) |
|  |  |  |  |  |  |  |
| **Duration of diabetes (years)** |  |  |  |  |  |  |
| Median (IQR) | 7(3-12) | | 7 (4-13) | | 7 (4-13) | |
| ≥0<5 years | 2918 | (33.0%) | 751 | (30.6%) | 1222 | (29.2%) |
| ≥5<10 years | 2544 | (28.8%) | 690 | (28.1%) | 1100 | (26.3%) |
| ≥10<20 years | 1952 | (22.1%) | 552 | (22.5%) | 1033 | (24.7%) |
| ≥20 years | 1020 | (11.5%) | 313 | (12.7%) | 529 | (12.7%) |
| Unknown | 412 | (4.7%) | 149 | (6.1%) | 295 | (7.1%) |
|  |  |  |  |  |  |  |
| **Diabetes management** |  |  |  |  |  |  |
| Diet only | 1508 | (17.0%) | 396 | (16.1%) | 625 | (15.0%) |
| Oral hypoglycaemic agent(s) only | 5166 | (58.4%) | 1416 | (57.7%) | 2438 | (58.3%) |
| Insulin +/- oral hypoglycaemic agent(s) | 2172 | (24.6%) | 643 | (26.2%) | 1116 | (26.7%) |
|  |  |  |  |  |  |  |
| **Participant-reported diabetic retinopathy** |  |  |  |  |  |  |
| Yes | 1628 | (18.4%) | 537 | (21.9%) | 858 | (20.5%) |
| No | 7151 | (80.8%) | 1895 | (77.2%) | 3267 | (78.2%) |
| Unknown | 67 | (0.8%) | 23 | (0.9%) | 54 | (1.3%) |
|  |  |  |  |  |  |  |
| **Participant-reported treatment for hypertension** |  |  |  |  |  |  |
| Yes | 5386 | (60.9%) | 1534 | (62.5%) | 2613 | (62.5%) |
| No | 3405 | (38.5%) | 904 | (36.8%) | 1526 | (36.5%) |
| Unknown | 55 | (0.6%) | 17 | (0.7%) | 40 | (1.0%) |
|  |  |  |  |  |  |  |
| **Systolic blood pressure**  **(mmHg)** |  |  |  |  |  |  |
| Mean (SD) | 135.8±14.9 | | 136.3±15.2 | | 136.8±15.9 | |
| <130 | 2023 | (22.9%) | 522 | (21.3%) | 849 | (20.3%) |
| ≥130<140 | 1822 | (20.6%) | 491 | (20.0%) | 778 | (18.6%) |
| ≥140 | 2626 | (29.7%) | 720 | (29.3%) | 1209 | (28.9%) |
| Unknown | 2375 | (26.8%) | 722 | (29.4%) | 1343 | (32.1%) |
|  |  |  |  |  |  |  |
| **Diastolic blood pressure**  **(mmHg)** |  |  |  |  |  |  |
| Mean (SD) | 77.4±9.2 | | 77.0±9.6 | | 76.4±9.9 | |
| <75 | 2371 | (26.8%) | 650 | (26.5%) | 1202 | (28.8%) |
| ≥75 <85 | 2788 | (31.5%) | 753 | (30.7%) | 1078 | (25.8%) |
| ≥85 | 1311 | (14.8%) | 326 | (13.3%) | 554 | (13.3%) |
| Unknown | 2376 | (26.9%) | 726 | (29.6%) | 1345 | (32.2%) |
|  |  |  |  |  |  |  |
| **Body mass index**  **(kg/m^2^)** |  |  |  |  |  |  |
| Mean (SD) | 30.6±6.1 | | 30.9±6.3 | | 30.9±6.5 | |
| <25 | 1313 | (14.8%) | 309 | (12.6%) | 627 | (15.0%) |
| ≥25, <30 | 3230 | (36.5%) | 901 | (36.7%) | 1398 | (33.5%) |
| ≥30<35 | 2429 | (27.5%) | 688 | (28.0%) | 1123 | (26.9%) |
| ≥35 | 1619 | (18.3%) | 472 | (19.2%) | 870 | (20.8%) |
| Unknown | 255 | (2.9%) | 85 | (3.5%) | 161 | (3.9%) |
|  |  |  |  |  |  |  |
| **Cigarette smoking** |  |  |  |  |  |  |
| Current | 555 | (6.3%) | 224 | (9.1%) | 500 | (12.0%) |
| Former | 3947 | (44.6%) | 1133 | (46.2%) | 1971 | (47.2%) |
| Never | 4243 | (48.0%) | 1067 | (43.5%) | 1667 | (39.9%) |
| Unknown | 101 | (1.1%) | 31 | (1.3%) | 41 | (1.0%) |
|  |  |  |  |  |  |  |
| **Non-study medication** |  |  |  |  |  |  |
| ACE-inhibitor or ARB | 5115 | (57.8%) | 1461 | (59.5%) | 2479 | (59.3%) |
| Aspirin use before screening | 3157 | (35.7%) | 879 | (35.8%) | 1472 | (35.2%) |
| Thiazide or related diuretic | 1639 | (18.5%) | 465 | (18.9%) | 853 | (20.4%) |
| Calcium channel blocker | 2088 | (23.6%) | 605 | (24.6%) | 1080 | (25.8%) |
| Statin | 6769 | (76.5%) | 1862 | (75.8%) | 3022 | (72.3%) |
|  |  |  |  |  |  |  |
| **Total cholesterol**  **(mmol/L)** |  |  |  |  |  |  |
| Mean (SD) | 4.1±0.9 | | 4.1±0.9 | | 4.2±0.9 | |
| <4 | 2681 | (30.3%) | 704 | (28.7%) | 1169 | (28.0%) |
| ≥4 <5 | 2246 | (25.4%) | 595 | (24.2%) | 954 | (22.8%) |
| ≥5 | 817 | (9.2%) | 226 | (9.2%) | 427 | (10.2%) |
| Not available | 3102 | (35.1%) | 930 | (37.9%) | 1629 | (39.0%) |
|  |  |  |  |  |  |  |
| **HDL cholesterol**  **(mmol/L)** |  |  |  |  |  |  |
| Mean (SD) | 1.3±0.4 | | 1.3±0.4 | | 1.2±0.4 | |
| <1 | 1222 | (13.8%) | 343 | (14.0%) | 605 | (14.5%) |
| ≥1<1.5 | 3202 | (36.2%) | 870 | (35.4%) | 1451 | (34.7%) |
| ≥1.5 | 1310 | (14.8%) | 307 | (12.5%) | 490 | (11.7%) |
| Not available | 3112 | (35.2%) | 935 | (38.1%) | 1633 | (39.1%) |
|  |  |  |  |  |  |  |
| **Non-HDL cholesterol**  **(mmol/L)** |  |  |  |  |  |  |
| Mean (SD) | 2.9±0.8 | | 2.9±0.8 | | 3.0±0.9 | |
| <2.5 | 2053 | (23.2%) | 505 | (20.6%) | 832 | (19.9%) |
| ≥2.5 <3.5 | 2558 | (28.9%) | 716 | (29.2%) | 1119 | (26.8%) |
| ≥3.5 | 1123 | (12.7%) | 299 | (12.2%) | 595 | (14.2%) |
| Not available | 3112 | (35.2%) | 935 | (38.1%) | 1633 | (39.1%) |
|  |  |  |  |  |  |  |
| **Glycosylated haemoglobin - HbA1c** |  |  |  |  |  |  |
| IFCC (mmol/mol) mean (SD) | 54.0±12.2 | | 55.6±13.5 | | 56.1±14.0 | |
| DCCT (%) mean (SD) | 7.1±1.1 | | 7.2±1.2 | | 7.3±1.3 | |
| <48 (6.5) | 1957 | (22.1%) | 496 | (20.2%) | 819 | (19.6%) |
| ≥48 (6.5), <64 (8.0) | 2792 | (31.6%) | 697 | (28.4%) | 1175 | (28.1%) |
| ≥64 (8.0) | 990 | (11.2%) | 332 | (13.5%) | 555 | (13.3%) |
| Not available | 3107 | (35.1%) | 930 | (37.9%) | 1630 | (39.0%) |
|  |  |  |  |  |  |  |
| **CKD-EPI estimated GFR**  **(ml/min/1.73m^2^)** |  |  |  |  |  |  |
| Mean (SD) | 87.8±19.8 | | 84.8±20.4 | | 79.5±23.1 | |
| ≥90 | 2902 | (32.8%) | 671 | (27.3%) | 950 | (22.7%) |
| ≥60<90 | 2299 | (26.0%) | 659 | (26.8%) | 1058 | (25.3%) |
| <60 | 540 | (6.1%) | 194 | (7.9%) | 542 | (13.0%) |
| Not available | 3105 | (35.1%) | 931 | (37.9%) | 1629 | (39.0%) |
|  |  |  |  |  |  |  |
| **Urinary albumin:creatinine ratio†**  **(mg/mmol)** |  |  |  |  |  |  |
| Median (IQR) | 0.50 (0.00-1.14) | | 0.59 (0.16-1.40) | | 0.71 (0.27-1.88) | |
| <3 | 5120 | (57.9%) | 1313 | (53.5%) | 2093 | (50.1%) |
| ≥3 | 613 | (6.9%) | 197 | (8.0%) | 438 | (10.5%) |
| Not available | 3113 | (35.2%) | 945 | (38.5%) | 1648 | (39.4%) |
|  |  |  |  |  |  |  |
| **Townsend Deprivation Index** |  |  |  |  |  |  |
| <-3 | 3101 | (35.1%) | 764 | (31.1%) | 1239 | (29.6%) |
| ≥-3<0 | 3588 | (40.6%) | 930 | (37.9%) | 1504 | (36.0%) |
| ≥0<2 | 1071 | (12.1%) | 364 | (14.8%) | 602 | (14.4%) |
| ≥2<4 | 625 | (7.1%) | 244 | (9.9%) | 446 | (10.7%) |
| ≥4<6 | 323 | (3.7%) | 112 | (4.6%) | 268 | (6.4%) |
| ≥6 | 118 | (1.3%) | 37 | (1.5%) | 106 | (2.5%) |
| Unknown | 20 | (0.2%) | 4 | (0.2%) | 14 | (0.3%) |
|  |  |  |  |  |  |  |
| **Ethnic origin** |  |  |  |  |  |  |
| White | 8564 | (96.8%) | 2346 | (95.6%) | 4025 | (96.3%) |
| Indian/Pakistani/Bangladeshi | 107 | (1.2%) | 39 | (1.6%) | 38 | (0.9%) |
| African/Caribbean | 54 | (0.6%) | 29 | (1.2%) | 57 | (1.4%) |
| Other/unknown | 121 | (1.4%) | 41 | (1.7%) | 59 | (1.4%) |
|  |  |  |  |  |  |  |

ACE= angiotensin-converting enzyme; ARB = angiotensin receptor blocker; DCCT = Diabetes Control and Complications Trial; FAs = Fatty acids; GFR = Glomerular Filtration Rate; HDL= High-density lipoprotein; IFCC = International Federation of Clinical Chemistry; IQR = Interquartile range; SD = Standard Deviation; VFQ=Visual Function Questionnaire

*Out of 8846 VFQ respondents, 7 answered a bespoke first page of questions that sought incident eye diagnoses but did not complete the NEI-VFQ-25, and 8839 completed both parts of the form.

†There was an analysis rule in ASCEND which stated that those with a below detectable threshold albumin component of their urinary albumin:creatinine ratio, would be recorded as zero. This applied to just over 25% of participants with no baseline eye screening records.

Percentages may not total 100 because of rounding.

## Table S3 Reported Definite or Probable Adherence with Study Treatment Stratified by Years Post-Randomisation

|  | **Aspirin Randomization** | | | **Omega-3 FAs Randomization** | | |
| --- | --- | --- | --- | --- | --- | --- |
|  | **Active (%)** | **Placebo (%)** | **Overall (%)** | **Active (%)** | **Placebo (%)** | **Overall (%)** |
| **Years post-randomisation** |  |  |  |  |  |  |
| <3 | 89.3% | 90.3% | 89.5% | 93.8% | 93.2% | 93.5% |
| ≥3<5 | 78.2% | 78.3% | 78.2% | 87.8% | 86.9% | 87.4% |
| ≥5<7 | 70.5% | 70.6% | 70.5% | 83.8% | 82.6% | 83.2% |
| ≥7 | 62.9% | 64.0% | 63.5% | 79.6% | 78.4% | 79.0% |
|  |  |  |  |  |  |  |
| **All** | **79.3%** | **79.3%** | **79.3%** | **88.4%** | **87.5%** | **87.9%** |

FAs=Fatty acids

Criteria used to define definite or probable adherence:

- Definitely adherent = participant reported taking treatment every or most days during the follow-up period.
- Probably adherent = participant was previously adherent, was still receiving medication and had not reported stopping treatment within the last seven months.

## Figure S1 General Vision Subdomain Score from the NEI-VFQ-25 by Aspirin Allocation


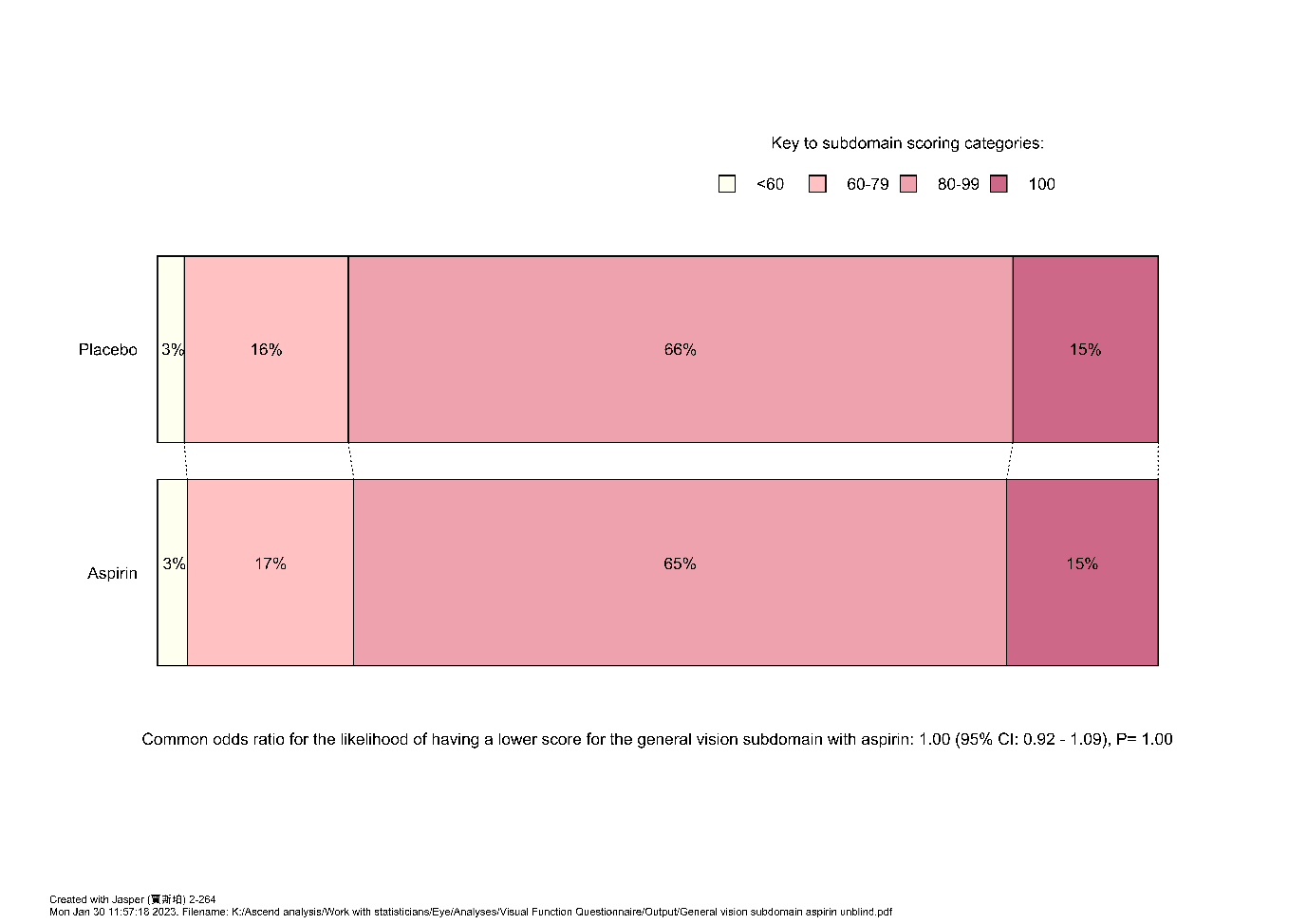


The number of participants who gave non-missing answers and contributed to this analysis was 8812

## Figure S2 General Vision Subdomain Score from the NEI-VFQ-25 by Omega-3 Fatty Acids Allocation


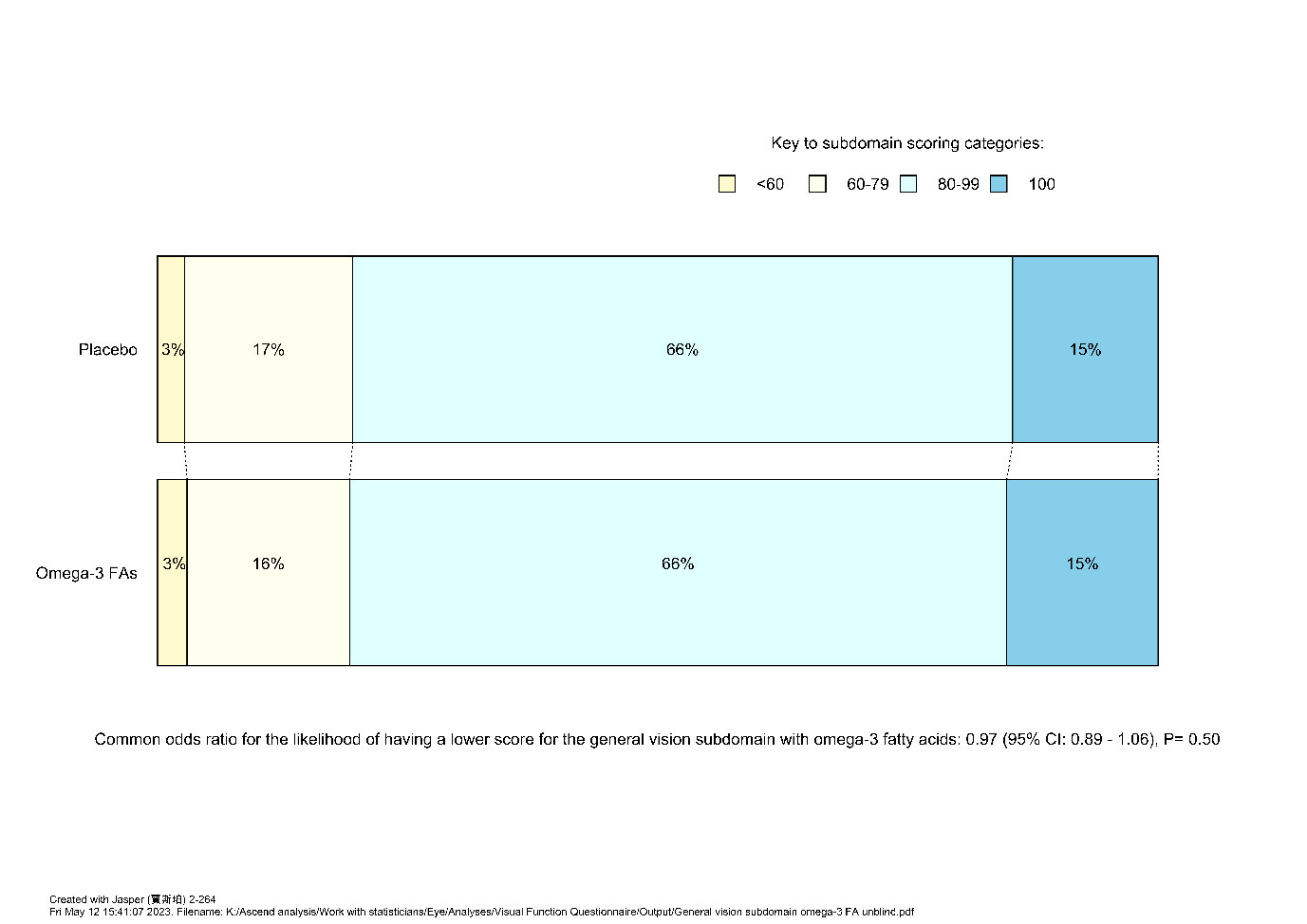


The number of participants who gave non-missing answers and contributed to this analysis was 8812

## Figure S3 Ocular Pain Subdomain Score from the NEI-VFQ-25 by Aspirin Allocation


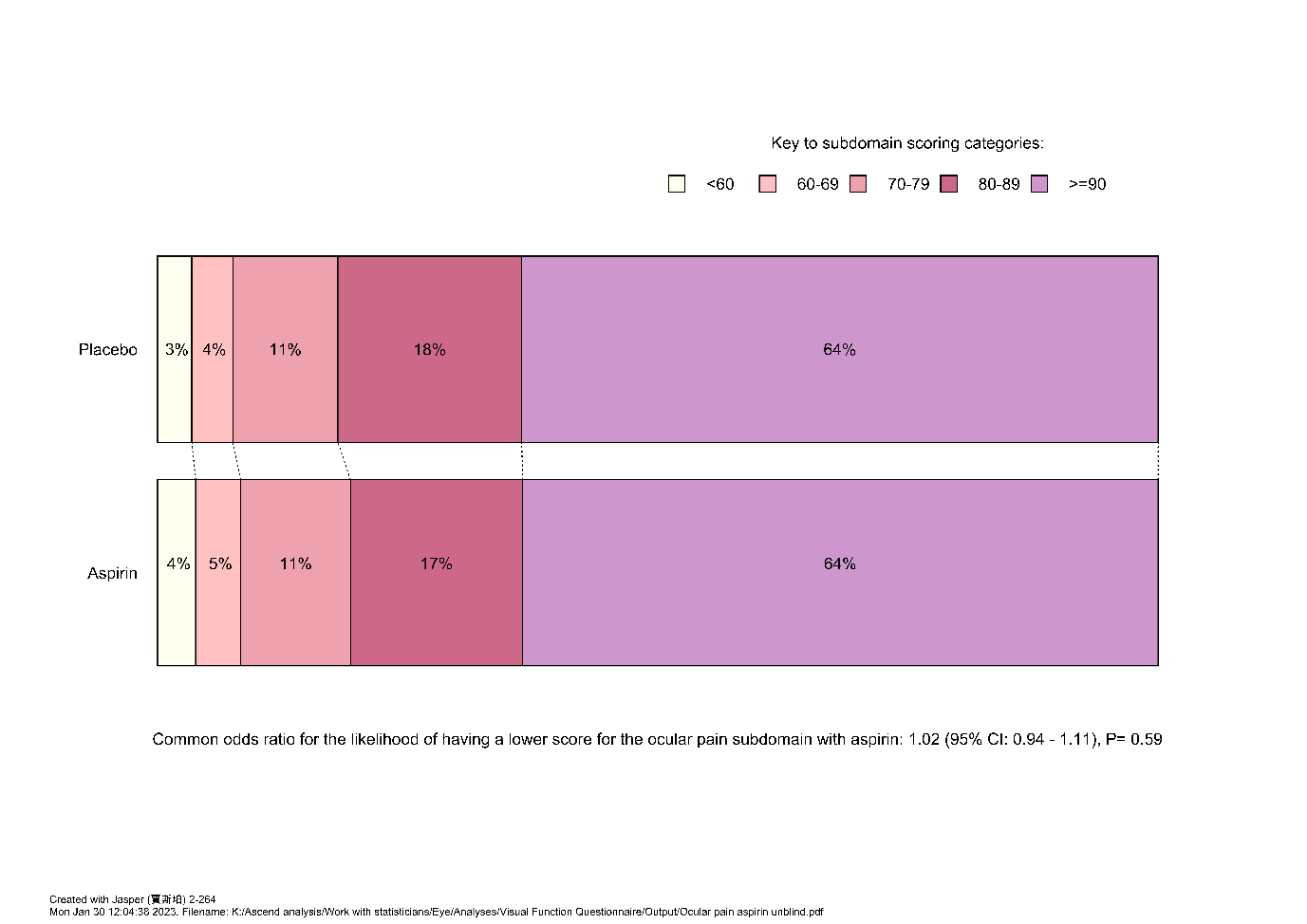


The number of participants who gave non-missing answers and contributed to this analysis was 8838

## Figure S4 Ocular Pain Subdomain Score from the NEI-VFQ-25 by Omega-3 Fatty Acids Allocation


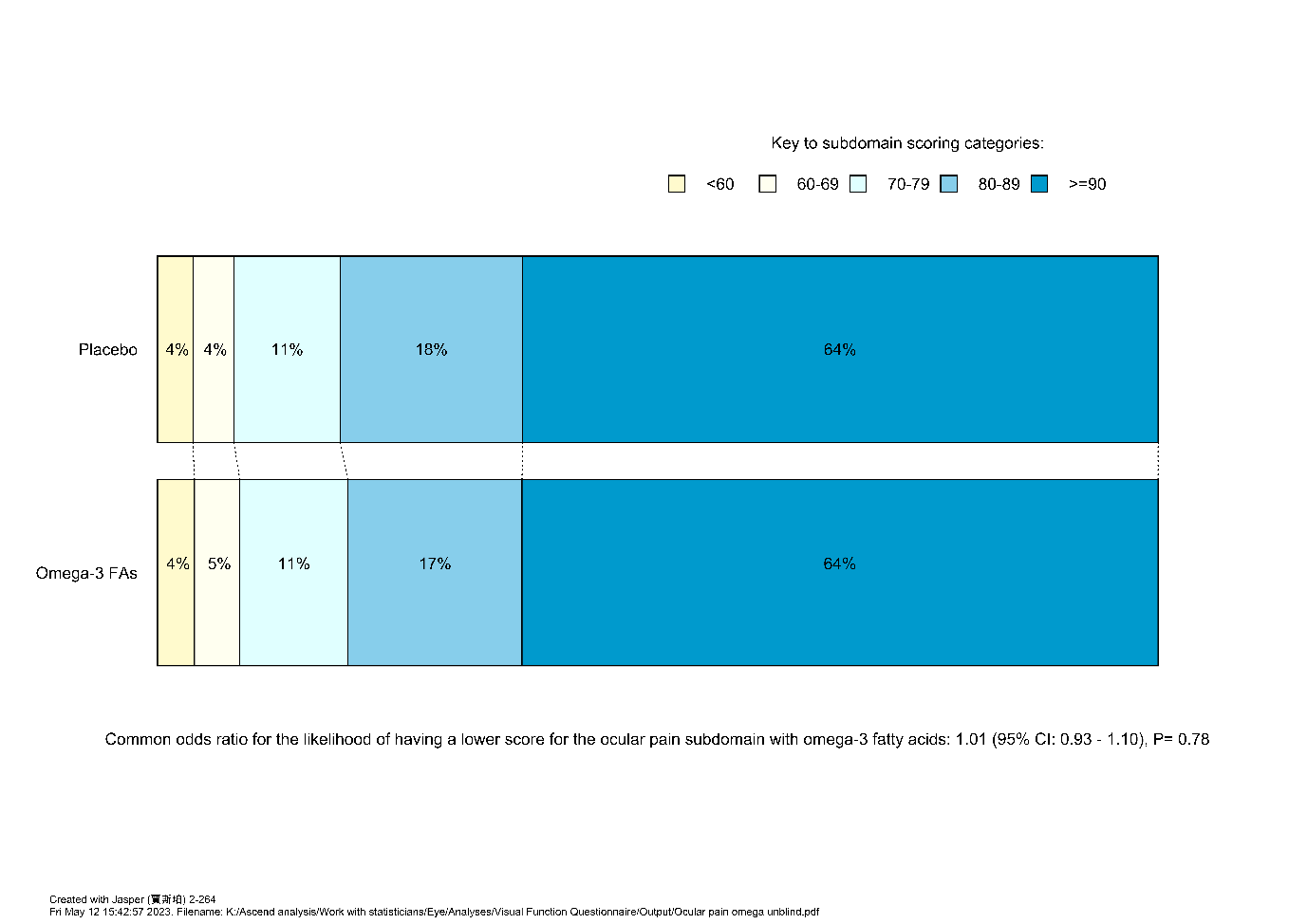


The number of participants who gave non-missing answers and contributed to this analysis was 8838

## Figure S5 Near Activities Subdomain Score from the NEI-VFQ-25 by Aspirin Allocation


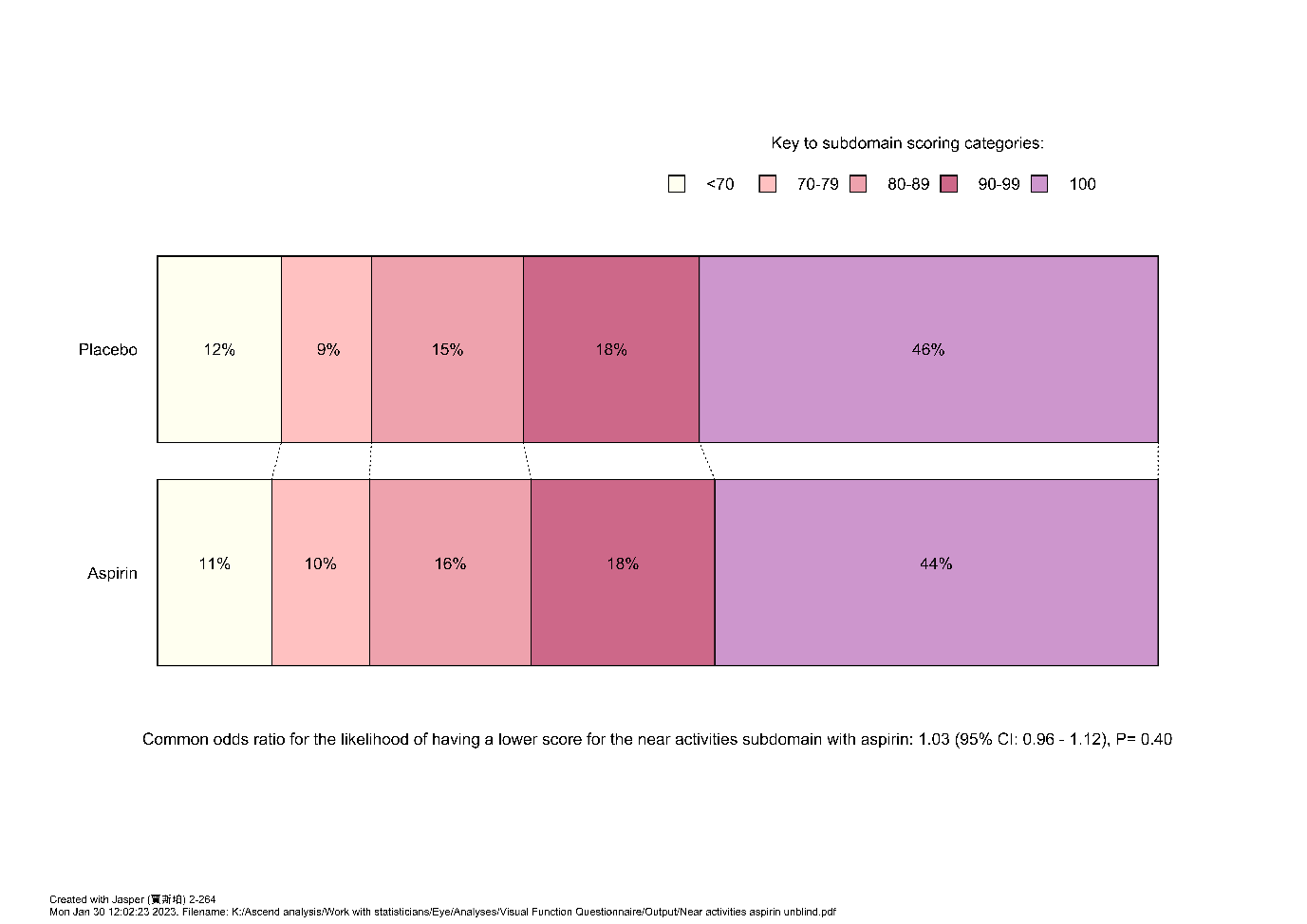


The number of participants who gave non-missing answers and contributed to this analysis was 8815

## Figure S6 Near Activities Subdomain Score from the NEI-VFQ-25 by Omega-3 Fatty Acids Allocation


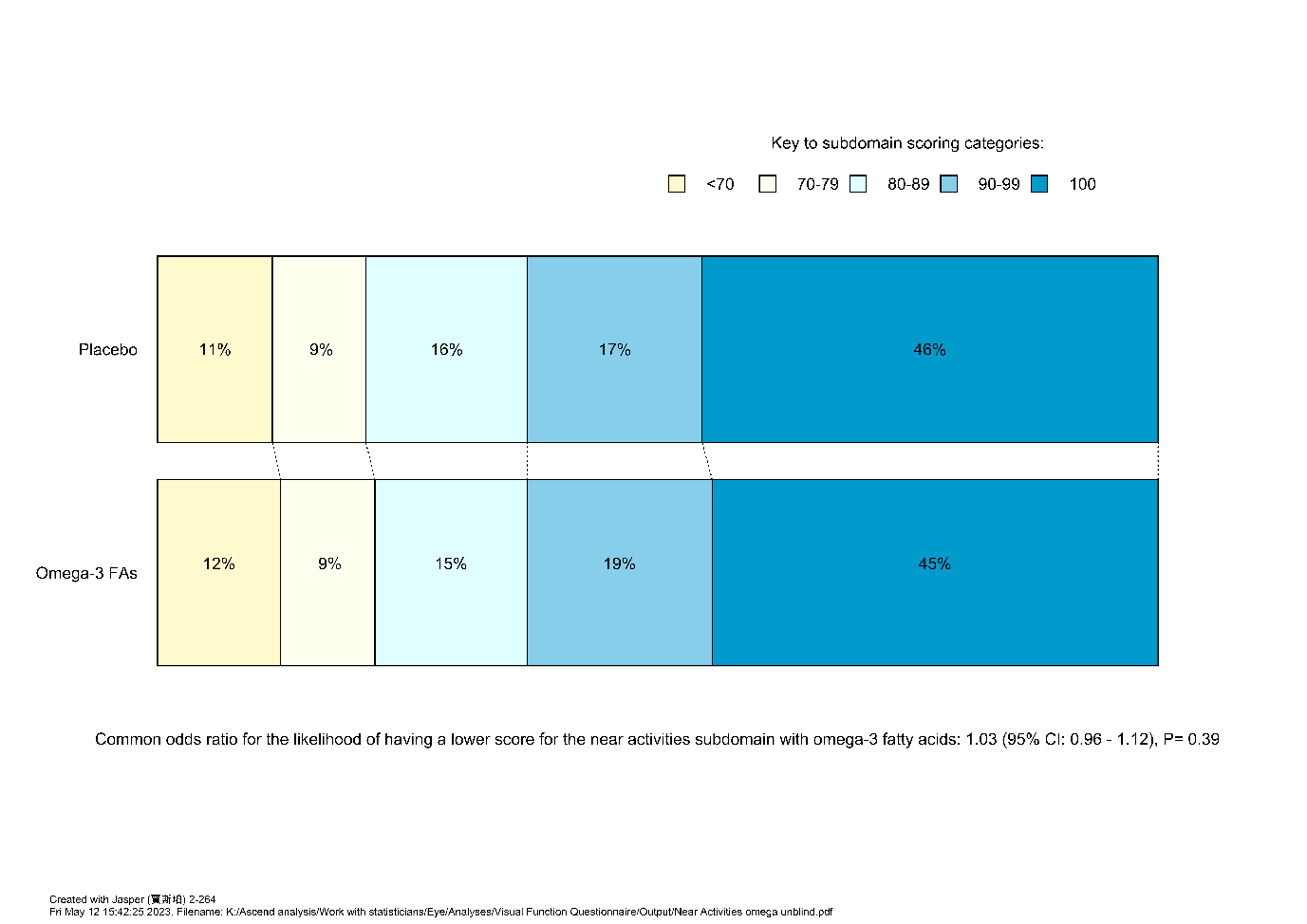


The number of participants who gave non-missing answers and contributed to this analysis was 8815

## Figure S7 Distance Activities Subdomain Score from the NEI-VFQ-25 by Aspirin Allocation


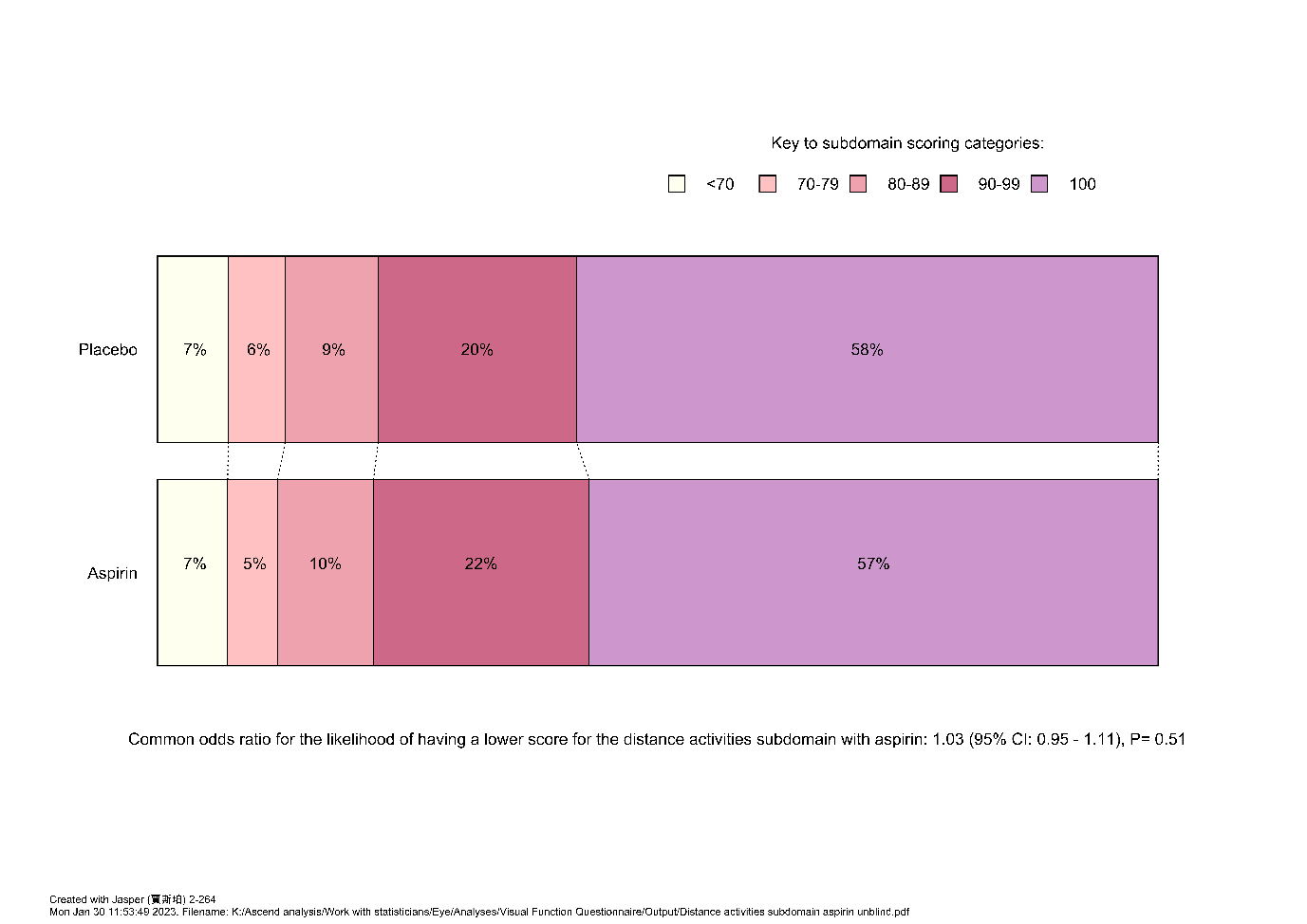


The number of participants who gave non-missing answers and contributed to this analysis was 8814

## Figure S8 Distance Activities Subdomain Score from the NEI-VFQ-25 by Omega-3 Fatty Acids Allocation


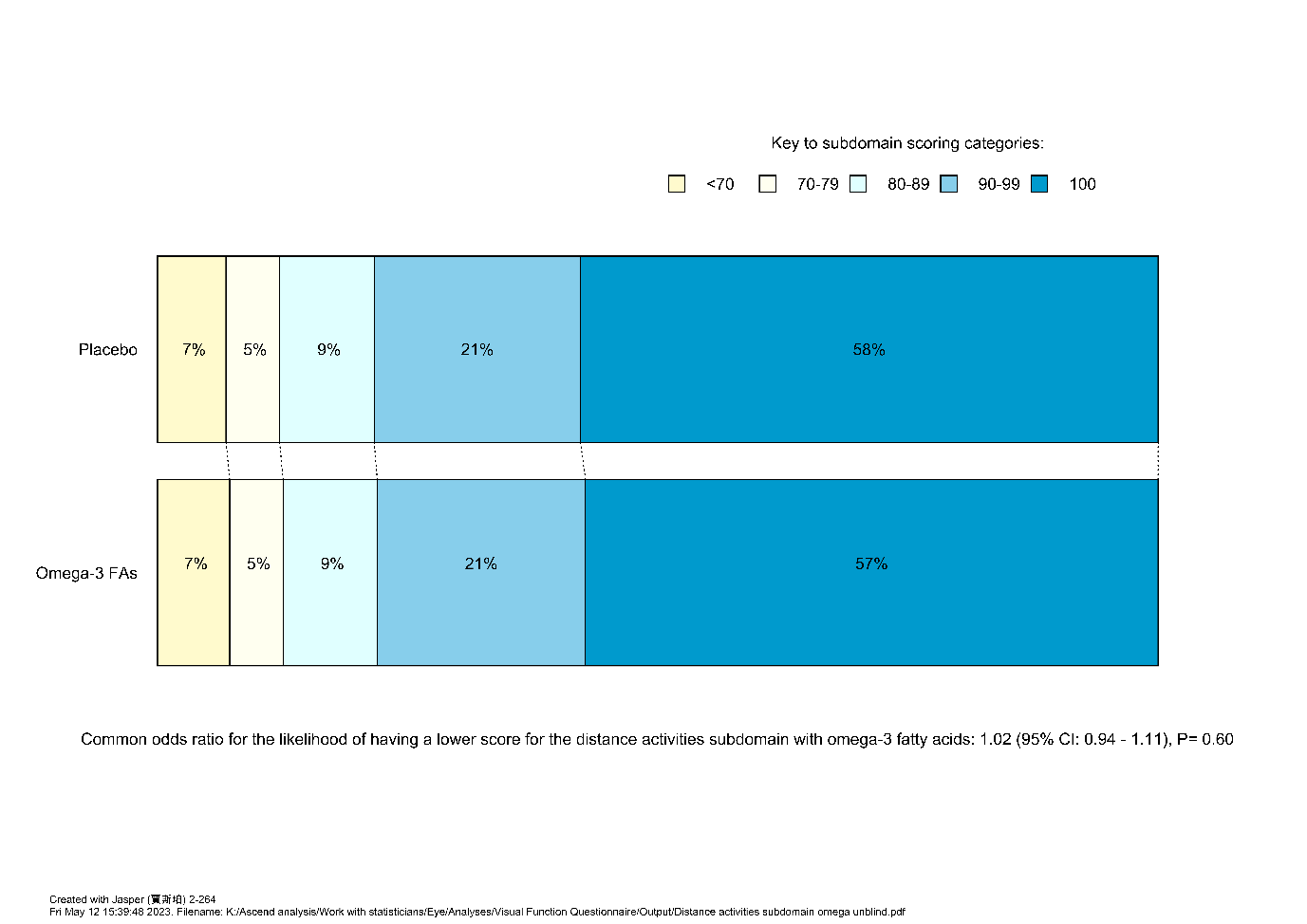


The number of participants who gave non-missing answers and contributed to this analysis was 8814

## Figure S9 Social Functioning Subdomain Score from the NEI-VFQ-25 by Aspirin Allocation


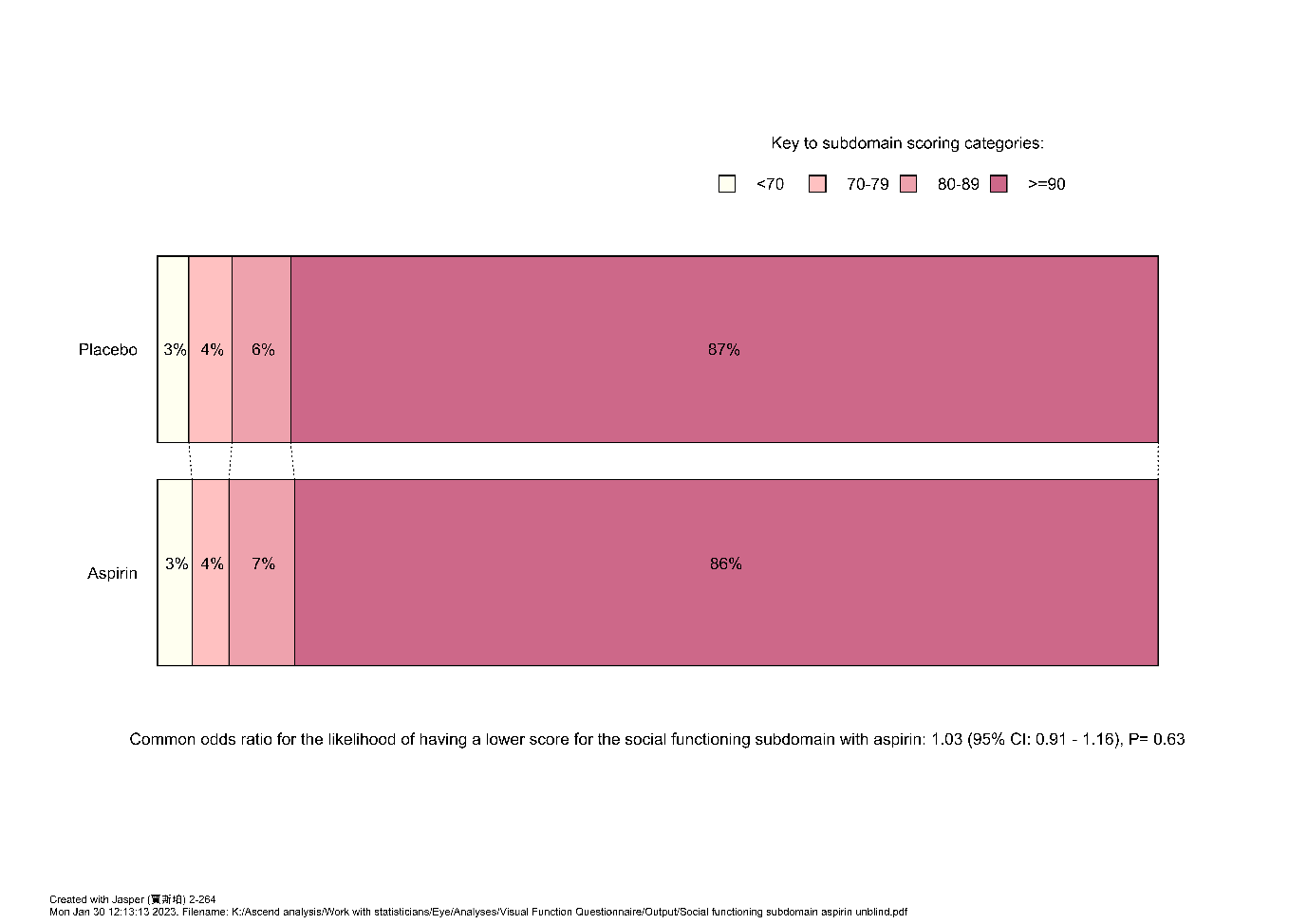


The number of participants who gave non-missing answers and contributed to this analysis was 8810

## Figure S10 Social Functioning Subdomain Score from the NEI-VFQ-25 by Omega-3 Fatty Acids Allocation


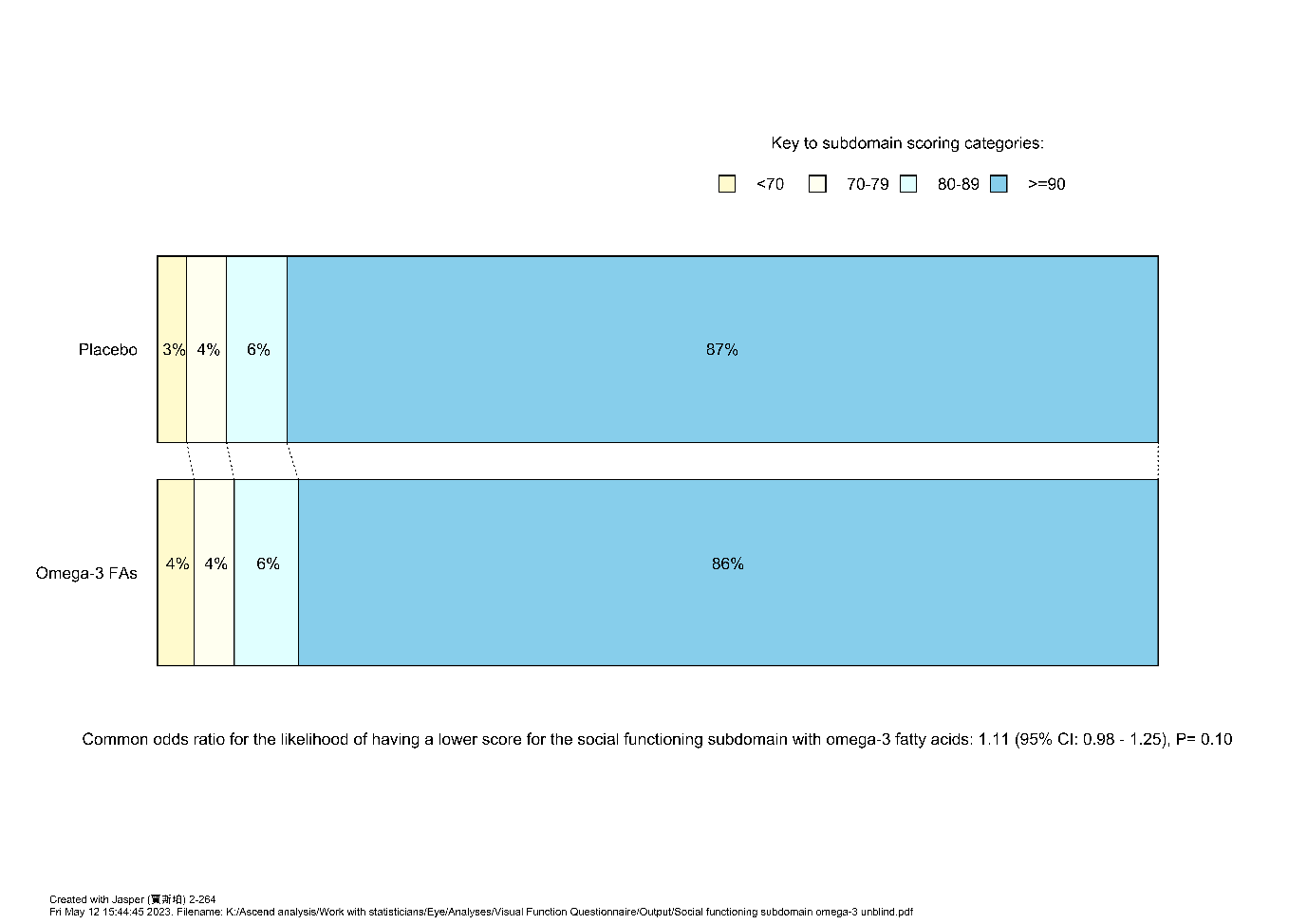


The number of participants who gave non-missing answers and contributed to this analysis was 8810

## Figure S11 Mental Health Subdomain Score from the NEI-VFQ-25 by Aspirin Allocation


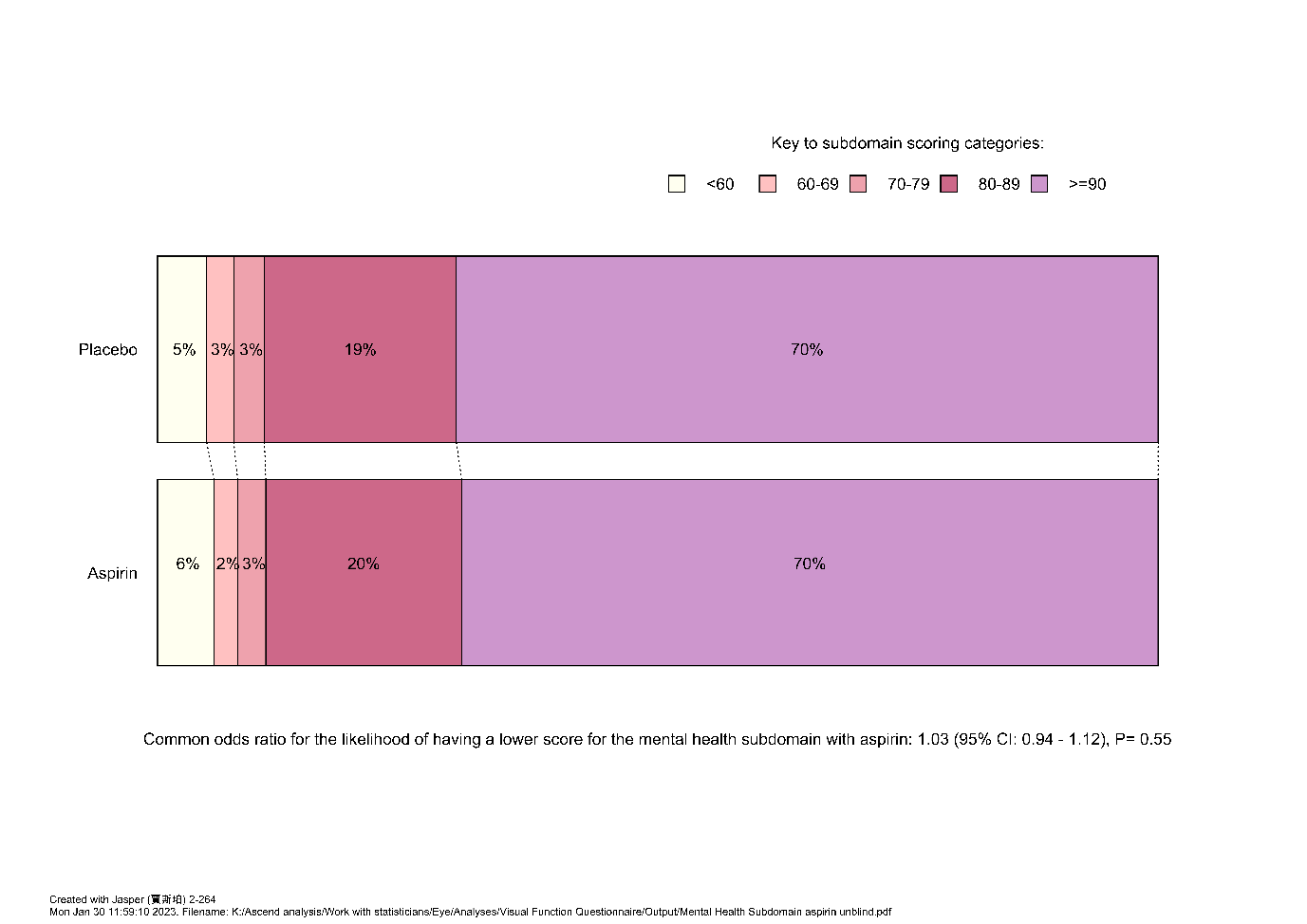


The number of participants who gave non-missing answers and contributed to this analysis was 8833

## Figure S12 Mental Health Subdomain Score from the NEI-VFQ-25 by Omega-3 Fatty Acids Allocation


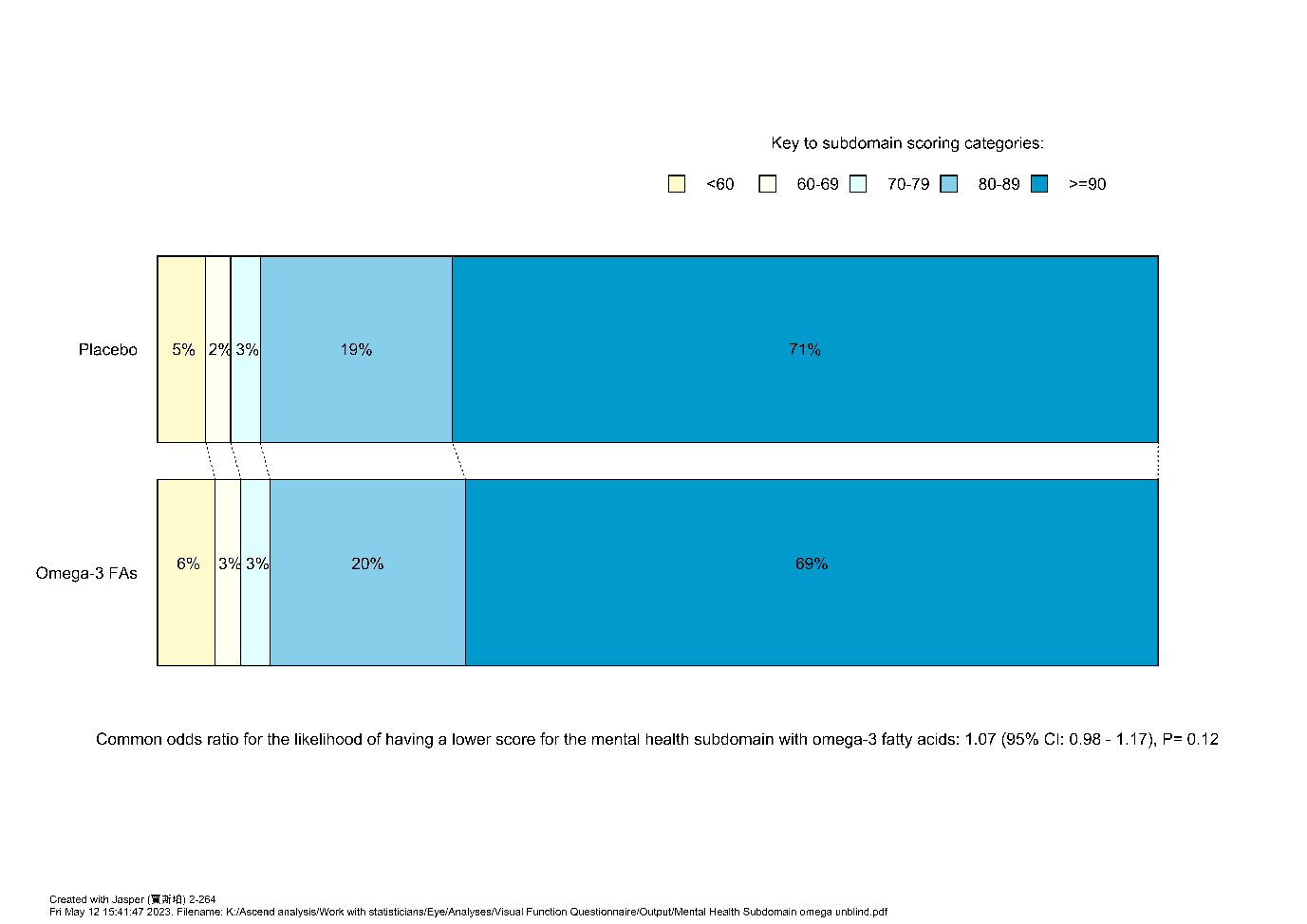


The number of participants who gave non-missing answers and contributed to this analysis was 8833

## Figure S13 Role Dependency Subdomain Score from the NEI-VFQ-25 by Aspirin Allocation


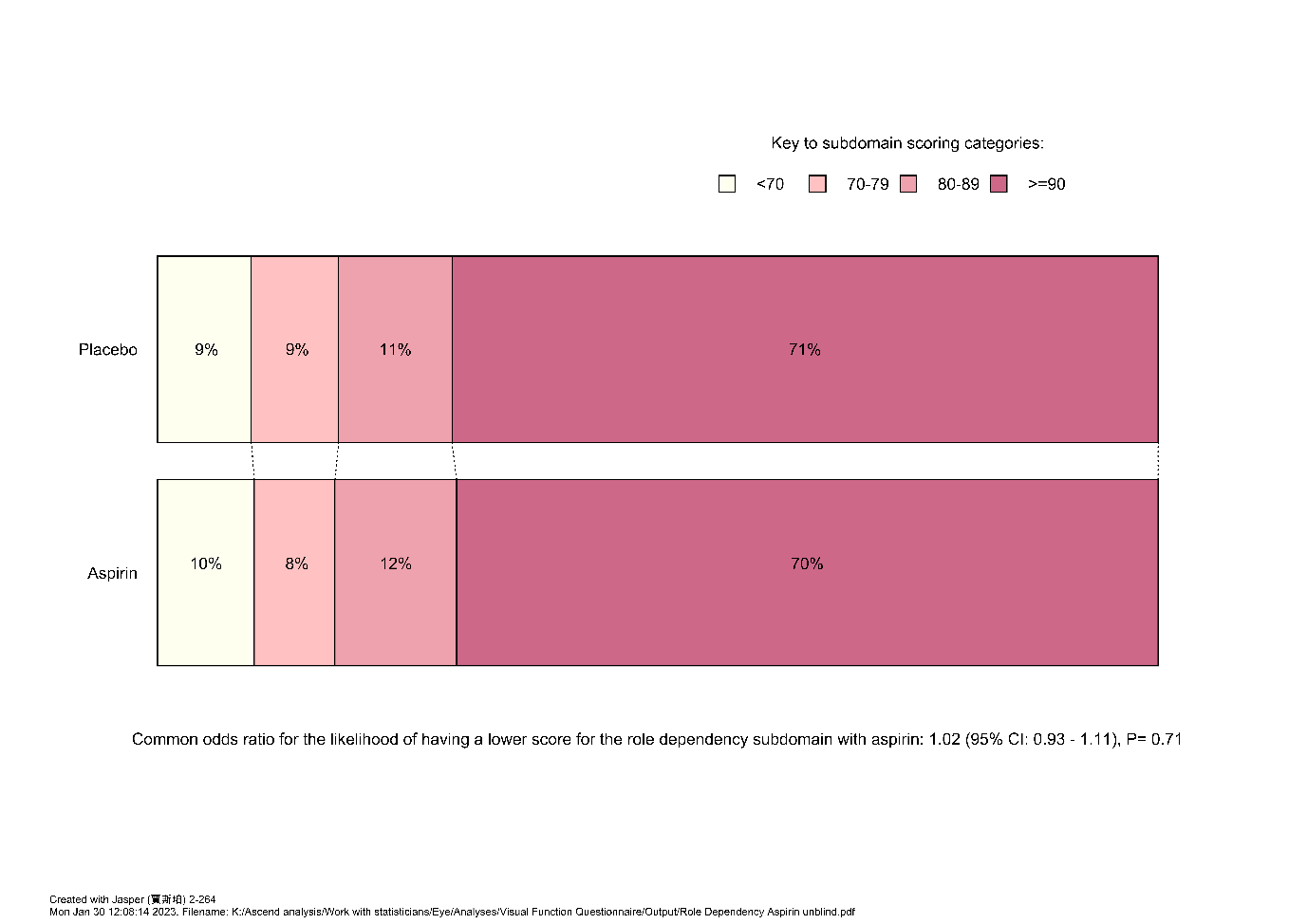


The number of participants who gave non-missing answers and contributed to this analysis was 8734

## Figure S14 Role Dependency Subdomain Score from the NEI-VFQ-25 by Omega-3 Fatty Acids Allocation


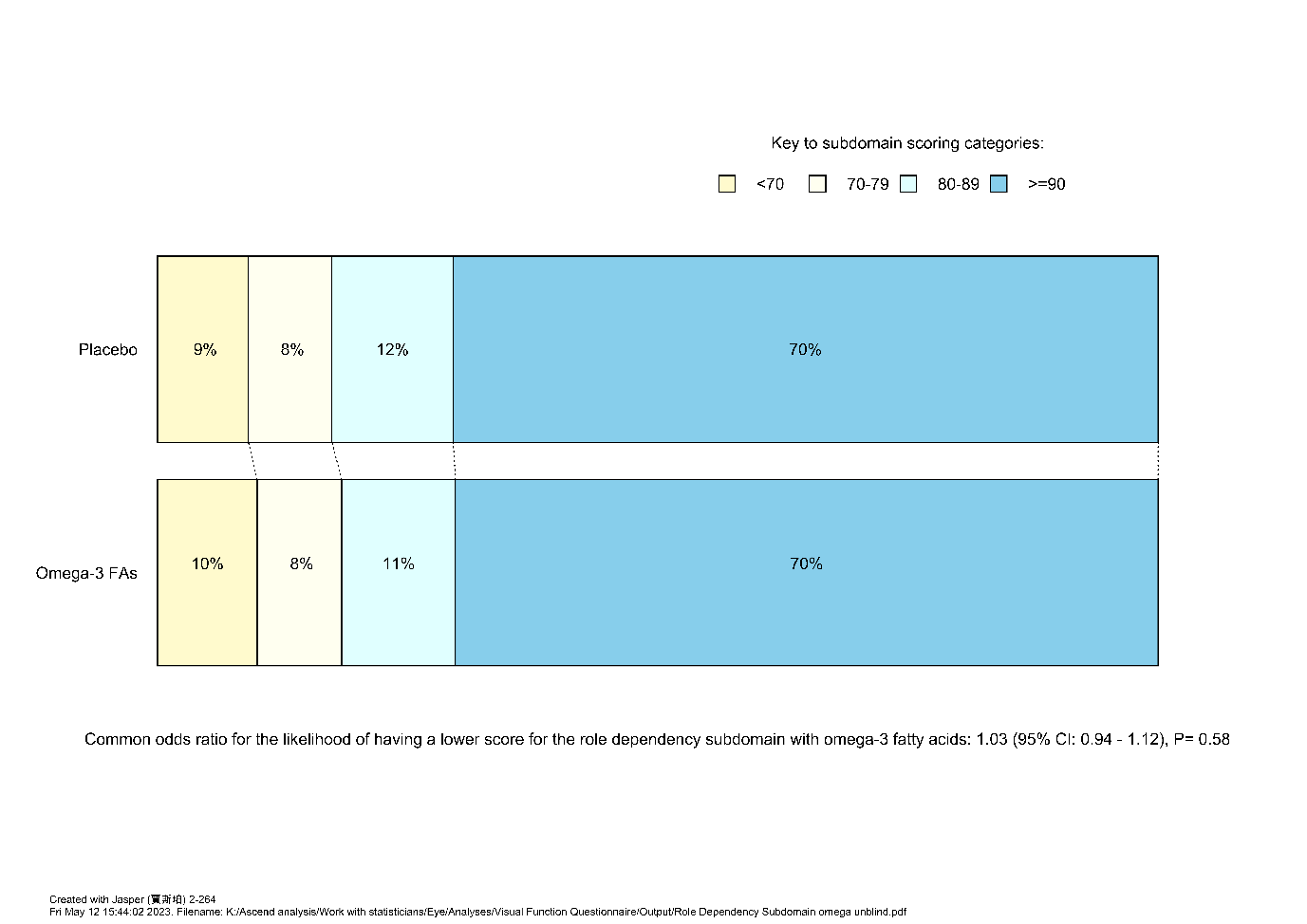


The number of participants who gave non-missing answers and contributed to this analysis was 8734

## Figure S15 Vision-Specific Dependency Subdomain Score from the NEI-VFQ-25 by Aspirin Allocation


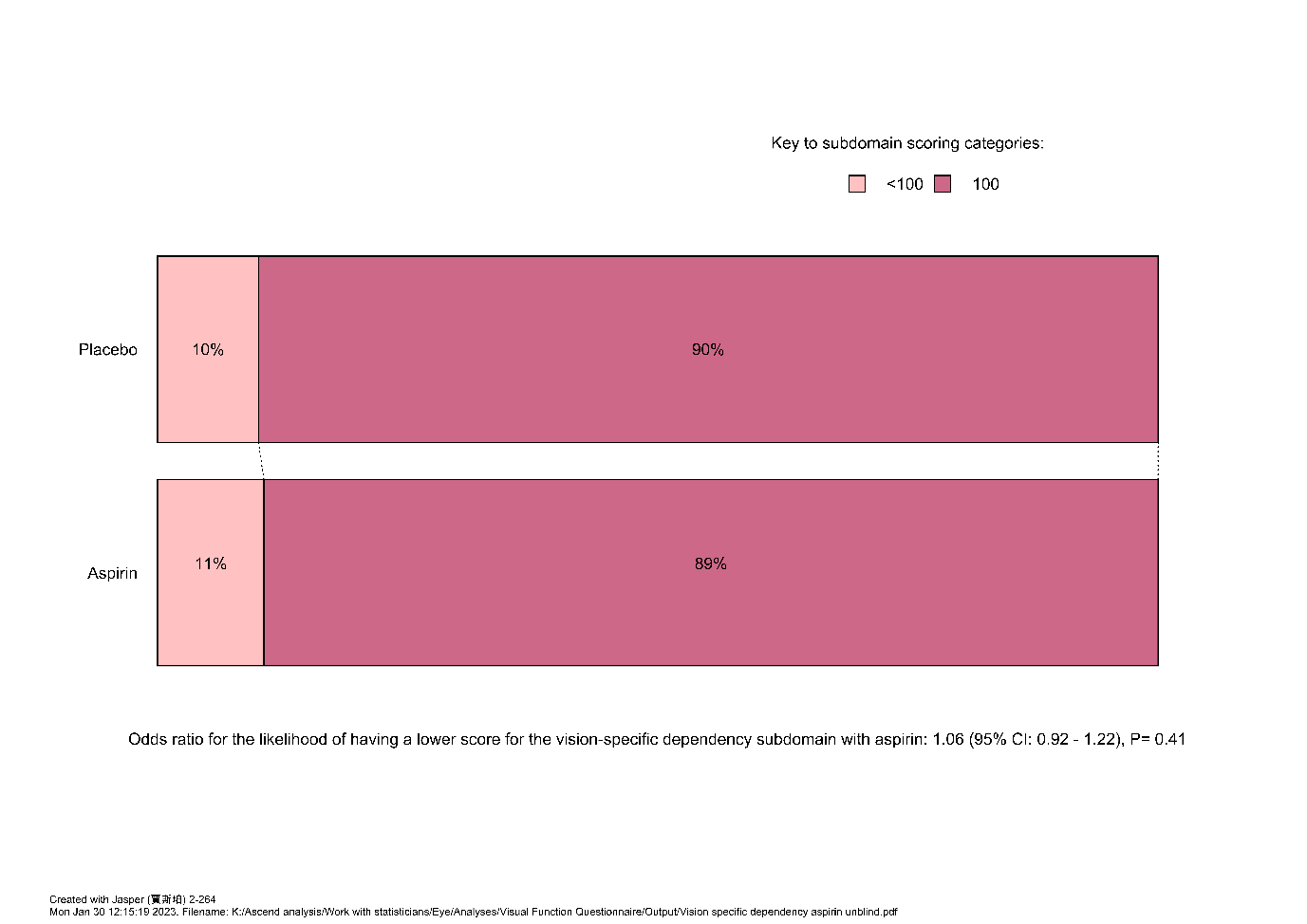


The number of participants who gave non-missing answers and contributed to this analysis was 8793

## Figure S16 Vision-Specific Dependency Subdomain Score from the NEI-VFQ-25 by Omega-3 Fatty Acids Allocation


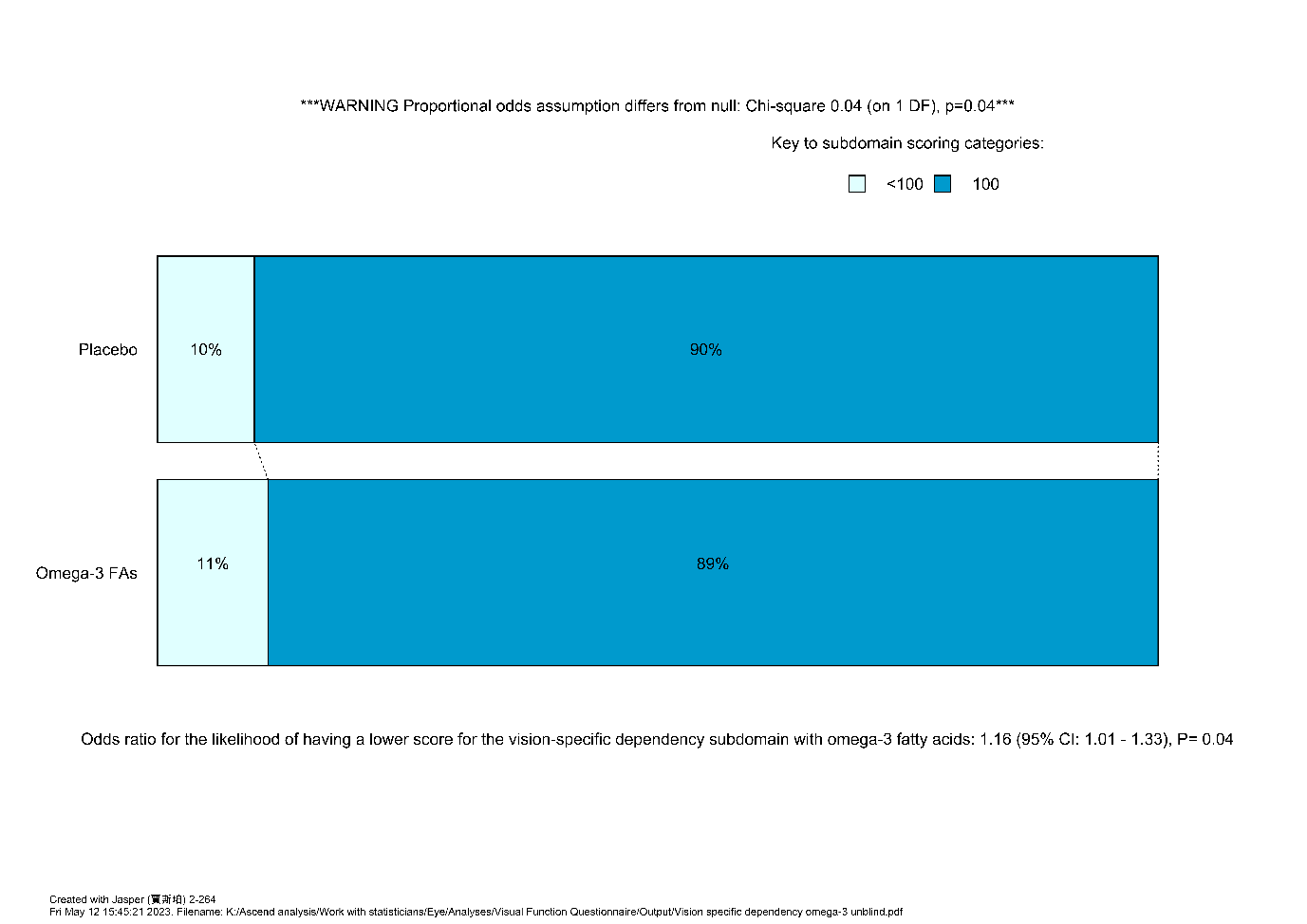


The number of participants who gave non-missing answers and contributed to this analysis was 8793

## Figure S17 Driving Subdomain Score from the NEI-VFQ-25 by Aspirin Allocation


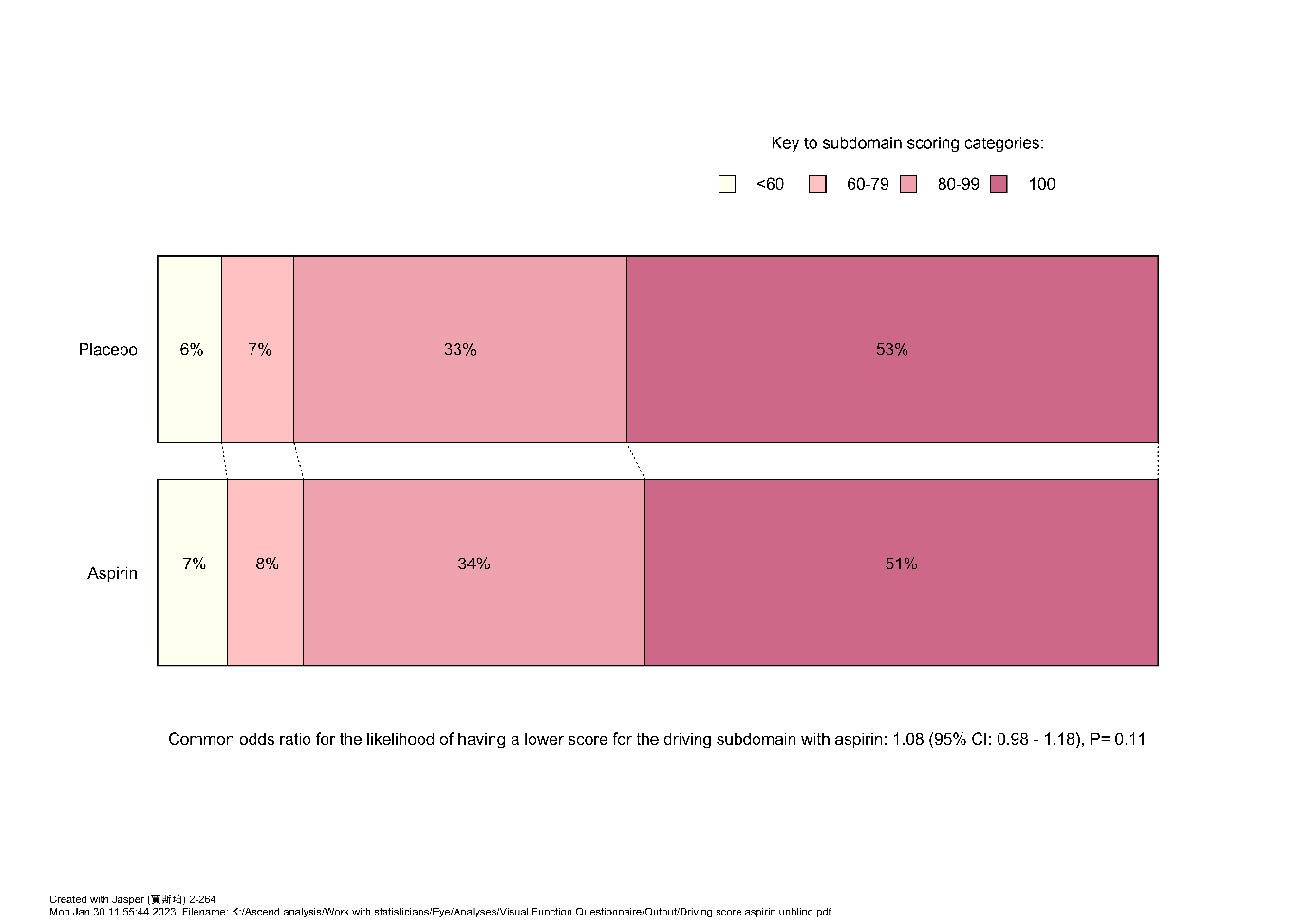


The number of participants who gave non-missing answers and contributed to this analysis was 6416

## Figure S18 Driving Subdomain Score from the NEI-VFQ-25 by Omega-3 Fatty Acids Allocation


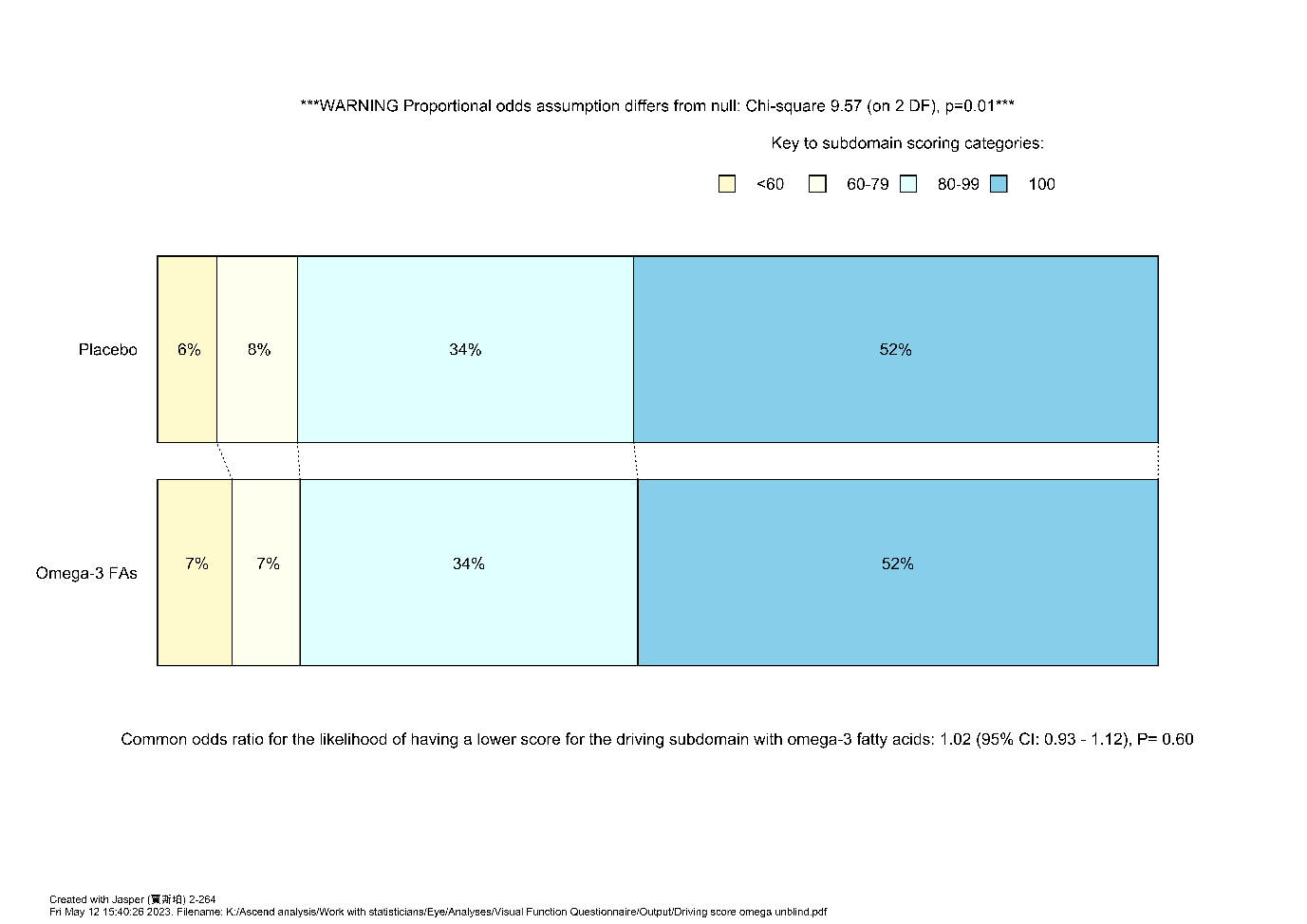


The number of participants who gave non-missing answers and contributed to this analysis was 6416

## Figure S19 Colour Vision Subdomain Score from the NEI-VFQ-25 by Aspirin Allocation


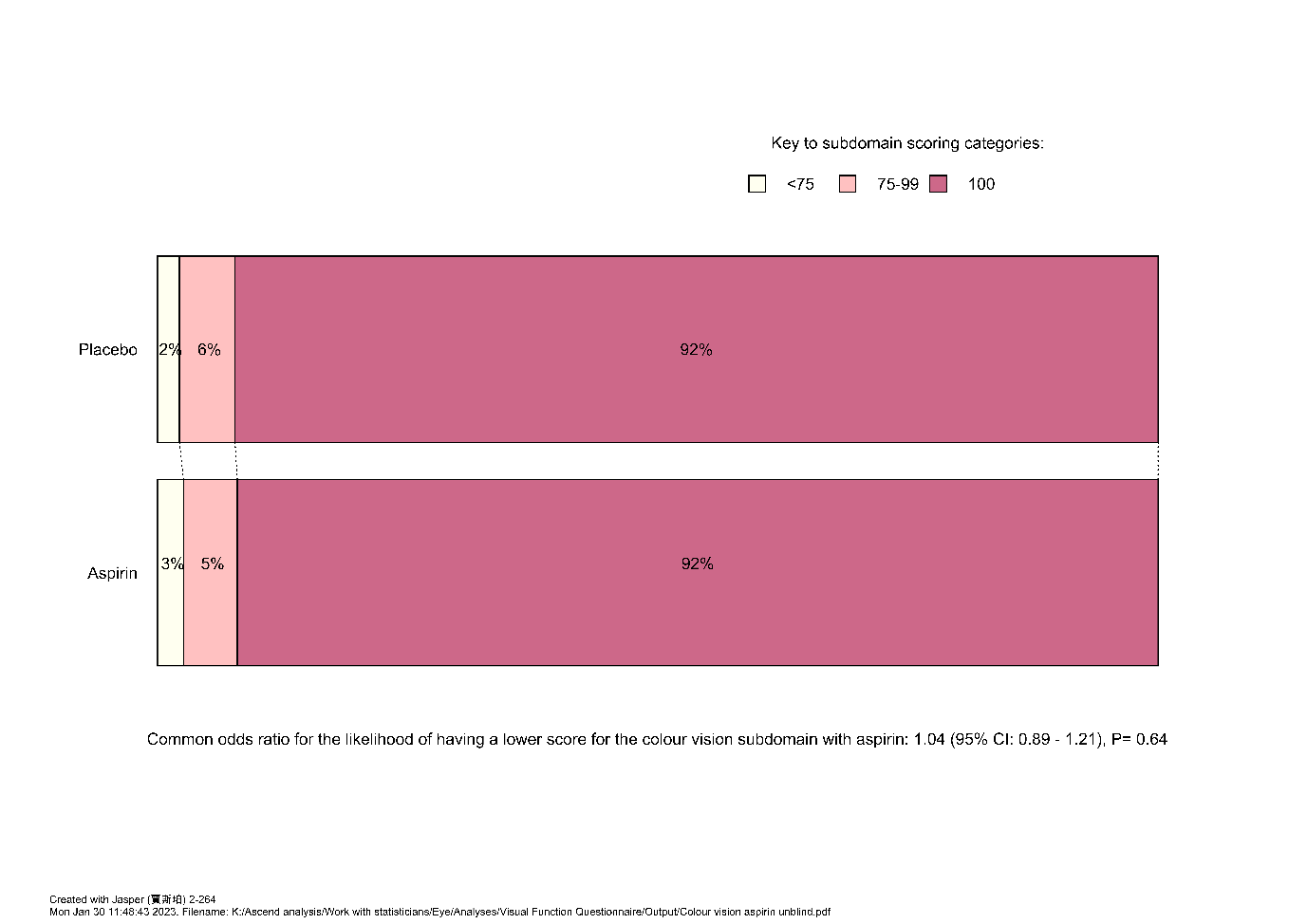


The number of participants who gave non-missing answers and contributed to this analysis was 8742

## Figure S20 Colour Vision Subdomain Score from the NEI-VFQ-25 by Omega-3 Fatty Acids Allocation


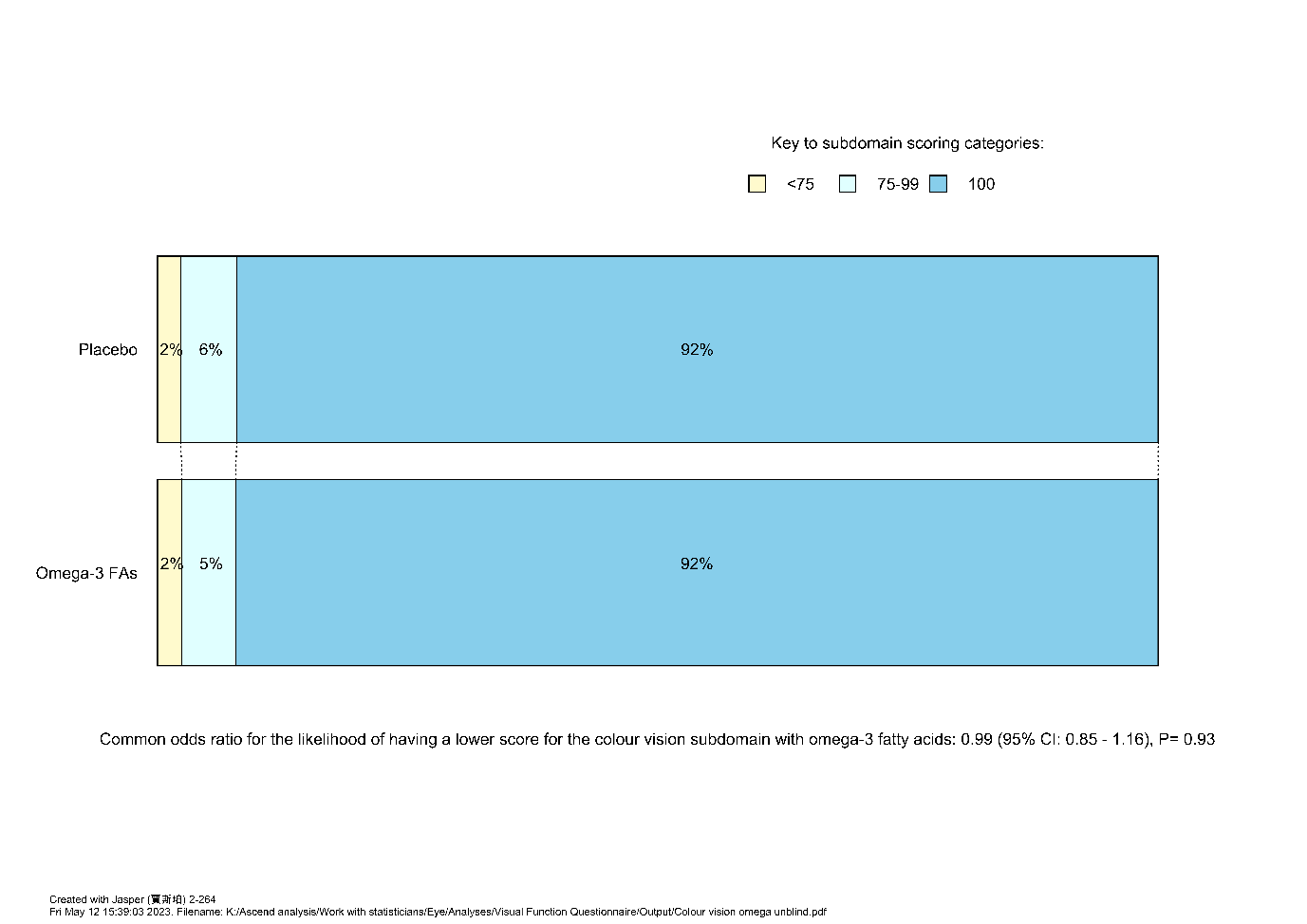


The number of participants who gave non-missing answers and contributed to this analysis was 8742

## Figure S21 Peripheral Vision Subdomain Score from the NEI-VFQ-25 by Aspirin Allocation


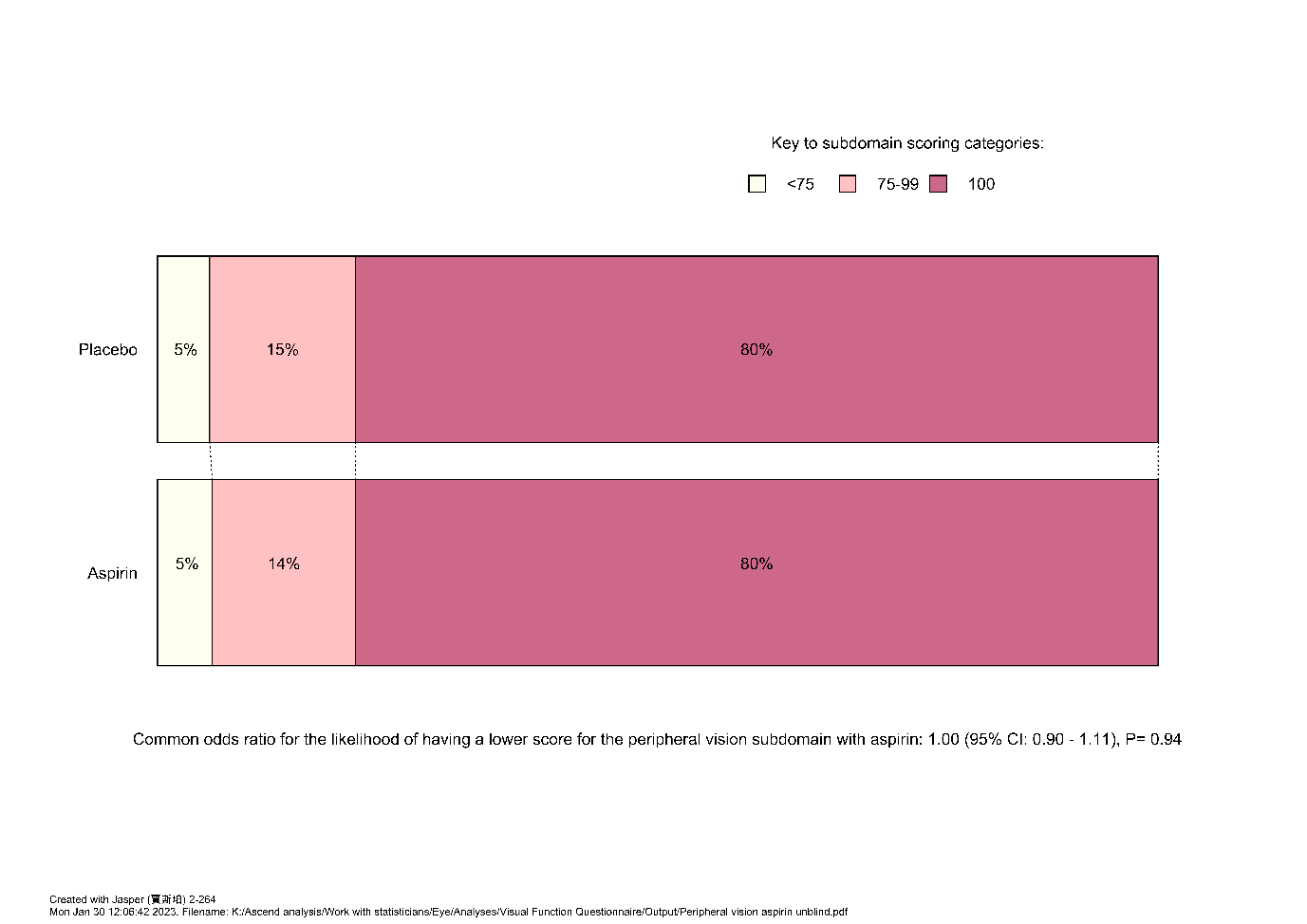


The number of participants who gave non-missing answers and contributed to this analysis was 8780

## Figure S22 Peripheral Vision Subdomain Score from the NEI-VFQ-25 by Omega-3 Fatty Acids Allocation


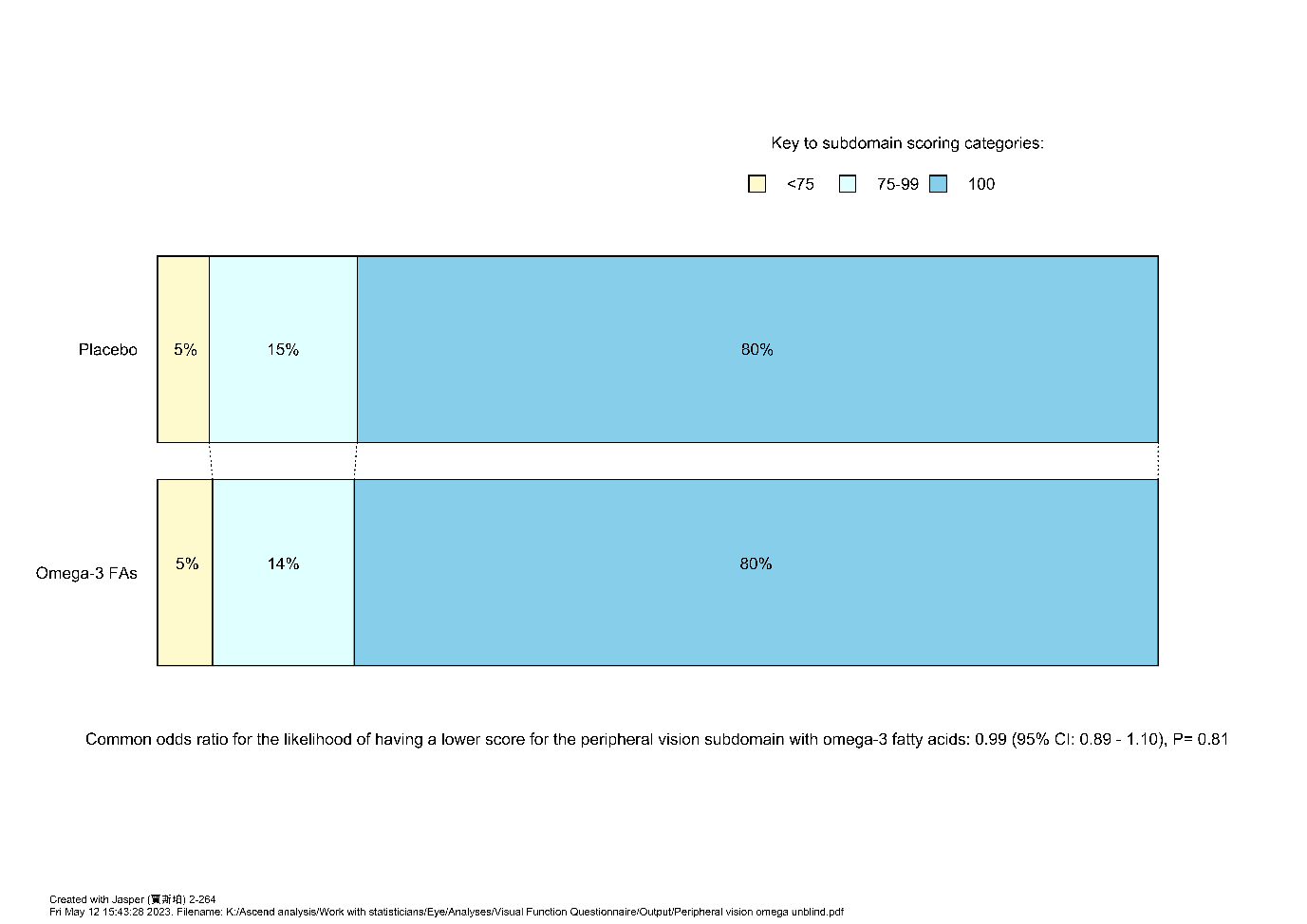


The number of participants who gave non-missing answers and contributed to this analysis was 8780

## Figure S23 General Health Subdomain Score from the NEI-VFQ-25 by Aspirin Allocation


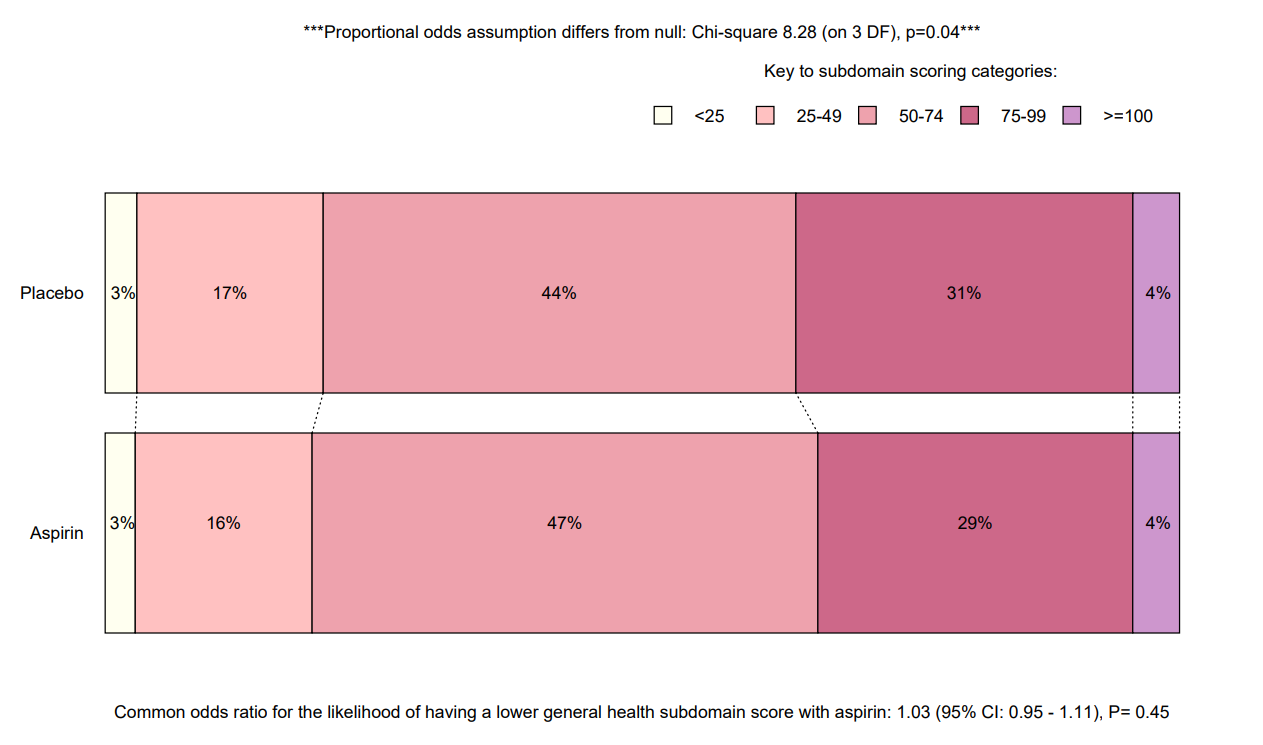


The number of participants who gave non-missing answers and contributed to this analysis was 8547

## Figure S24 General Health Subdomain Score from the NEI-VFQ-25 by Omega-3 Fatty Acids Allocation


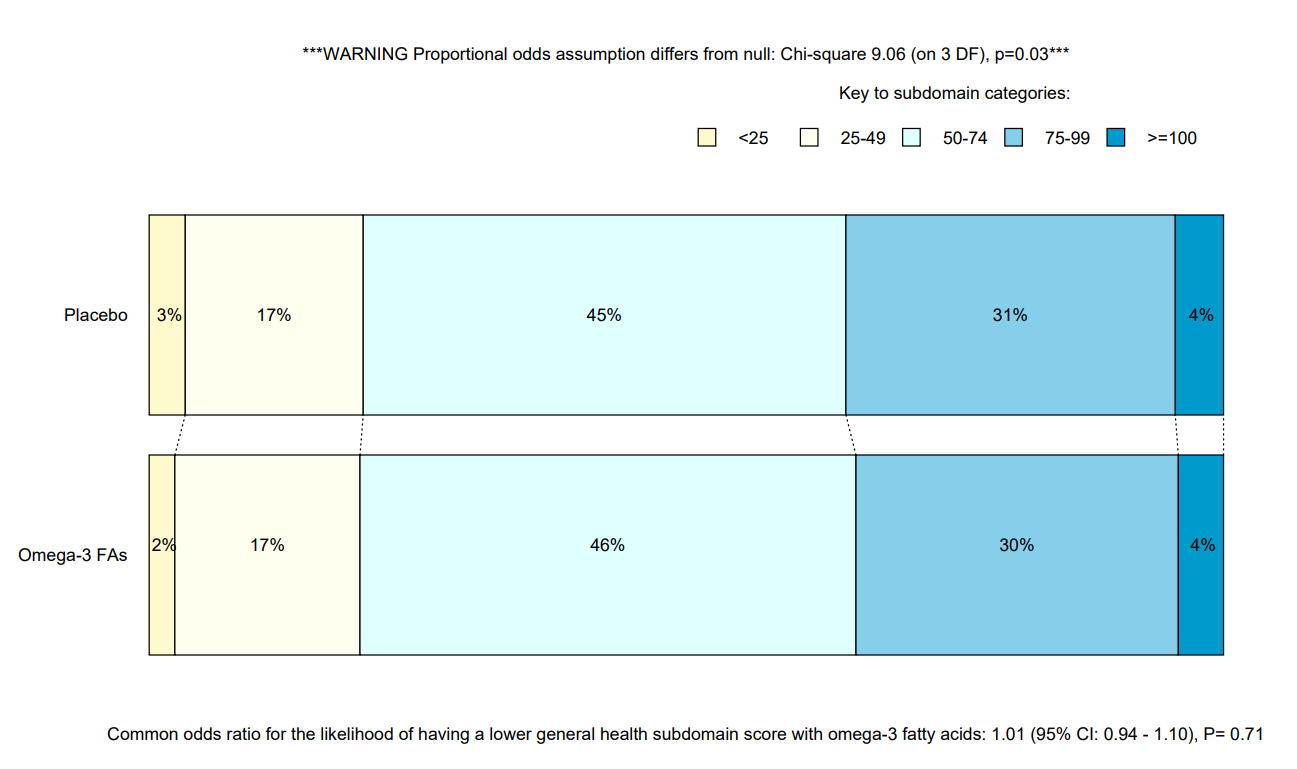


The number of participants who gave non-missing answers and contributed to this analysis was 8547

## References

1. Mangione CM, Lee PP, Gutierrez PR, *et al.* Development of the 25-list-item National Eye Institute Visual Function Questionnaire. *Archives of Ophthalmology* 2001; 119(7): 1050-8.

2. Roozenbeek B, Lingsma HF, Perel P*, et al.* The added value of ordinal analysis in clinical trials: an example in traumatic brain injury. *Critical Care* 2011; 15(3): R127.

3. Valenta Z, Pitha J, Poledne R. Proportional odds logistic regression--effective means of dealing with limited uncertainty in dichotomizing clinical outcomes. *Statistics in Medicine* 2006; 25(24): 4227-34.
